# *SF3B1* mutations provide genetic vulnerability to copper ionophores in human acute myeloid leukemia

**DOI:** 10.1126/sciadv.adl4018

**Published:** 2024-03-22

**Authors:** Céline Moison, Deanne Gracias, Julie Schmitt, Simon Girard, Jean-François Spinella, Simon Fortier, Isabel Boivin, Rodrigo Mendoza-Sanchez, Bounkham Thavonekham, Tara MacRae, Nadine Mayotte, Eric Bonneil, Mark Wittman, James Carmichael, Réjean Ruel, Pierre Thibault, Josée Hébert, Anne Marinier, Guy Sauvageau

**Affiliations:** ^1^ Institute for Research in Immunology and Cancer, Université de Montréal, Montréal, Canada.; ^2^ Research and Development, Bristol Myers Squibb Company, Cambridge, MA, USA.; ^3^ Department of Chemistry, Université de Montréal, Montréal, Canada.; ^4^ Division of Hematology-Oncology and Quebec Leukemia Cell Bank, Maisonneuve-Rosemont Hospital, Montréal, Canada.; ^5^ Department of Medicine, Faculty of Medicine, Université de Montréal, Montréal, Canada.

## Abstract

In a phenotypical screen of 56 acute myeloid leukemia (AML) patient samples and using a library of 10,000 compounds, we identified a hit with increased sensitivity toward *SF3B1*-mutated and adverse risk AMLs. Through structure-activity relationship studies, this hit was optimized into a potent, specific, and nongenotoxic molecule called UM4118. We demonstrated that UM4118 acts as a copper ionophore that initiates a mitochondrial-based noncanonical form of cell death known as cuproptosis. CRISPR-Cas9 loss-of-function screen further revealed that iron-sulfur cluster (ISC) deficiency enhances copper-mediated cell death. Specifically, we found that loss of the mitochondrial ISC transporter *ABCB7* is synthetic lethal to UM4118. *ABCB7* is misspliced and down-regulated in *SF3B1*-mutated leukemia, creating a vulnerability to copper ionophores. Accordingly, ABCB7 overexpression partially rescued *SF3B1*-mutated cells to copper overload. Together, our work provides mechanistic insights that link ISC deficiency to cuproptosis, as exemplified by the high sensitivity of *SF3B1*-mutated AMLs. We thus propose *SF3B1* mutations as a biomarker for future copper ionophore–based therapies.

## Teaser

Missplicing of the iron-sulfur cluster transporter *ABCB7*, found in *SF3B1*-mutated AML, sensitizes cells to cuproptosis.

## INTRODUCTION

Copper is an essential transition metal for all living organisms (*1*). It acts as a cofactor in multiple enzymatic reactions due to its redox properties which enhance catalytic functions (*2*). Dysregulation of intracellular copper concentration is highly detrimental, as exemplified by Wilson’s and Menke’s diseases, two life-threatening genetic disorders in which mutations in copper transporter cause intracellular copper accumulation or deprivation, respectively. In cancer, increased copper levels have been reported both in serum and in tumor cells and are associated with enhanced proliferation and tumor burden (*3*–*6*). Therapeutic approaches have thus emerged to disrupt copper homeostasis by using either copper chelators or ionophores (*7*). Copper ionophores are copper-binding small molecules capable to cross the plasma membrane, causing intracellular copper accumulation and ultimately triggering cuproptosis, a noncanonical cell death program (*8*).

Insights into the mechanism involved in cuproptosis were proposed in a recent publication (*8*), where the functional link between copper overload and its impact on cellular respiration was reported. The authors found that copper-induced cell death primarily involves the binding of this cation to lipoylated enzymes [e.g., Dihydrolipoamide S-Acetyltransferase (DLAT), Dihydrolipoamide  S-Succinyltransferase (DLST), etc.] that regulate carbon entry points in the tricarboxylic acid (TCA) cycle. Lipoylation is essential for the activity of these enzymes and is increased in cells that depend on oxidative phosphorylation (OXPHOS), in which the TCA cycle is the key reduced form of nicotinamide adenine dinucleotide (NADH) donor for electron transfer chain (ETC) activity. Copper binding to lipoylated proteins generates aggregation and leads to proteotoxic stress. In addition, the authors also noticed that another class of essential proteins, referred to as the iron-sulfur (Fe-S) cluster (ISC)–containing proteins, is depleted upon exposure to elesclomol, a known copper ionophore. However, mechanistic links are missing regarding how copper affects ISC-containing proteins stability and how the latter participates to cuproptosis.

ISC are a series of inorganic protein cofactors strictly assembled in the mitochondrial matrix by a specialized machinery called the ISC assembly machinery (*9*, *10*). ISCs are necessary for numerous proteins, including the ETCI-III complexes, where they promote electron transfer, the lipoylation enzyme Lipoic Acid Synthetase (LIAS), and others involved in DNA synthesis, DNA repair, iron regulation, nucleotide metabolism, and ribosome biogenesis (*11*). ISCs are exported to the cytosol through a transporter, putatively ABCB7 (ATP binding cassette subfamily B member 7), where they are loaded onto proteins by the cytosolic iron-sulfur assembly (CIA) machinery (*12*). Deficiency of the ABCB7 transporter impairs the maturation of cytosolic ISC enzymes and cause X-linked sideroblastic anemia with ataxia (*13*–*15*).

Acute myeloid leukemia (AML) is a heterogeneous disease with outcomes highly dependent on cytogenetics and mutational profiles. With the aim of identifying advanced therapeutic strategies to improve survival of poor-prognosis AML patients, we conducted a high-throughput chemical screening on a panel of genetically diverse primary AML specimens. We identified, optimized, and characterized copper ionophore molecules that showed heightened activity against poor-prognosis AMLs, in particular those carrying splicing factor 3b subunit 1 (*SF3B1*) mutations.

*SF3B1* is the most frequently mutated splicing gene in cancer, leading to missplicing of numerous mRNAs due to aberrant recognition of branch point sequences (*16*, *17*). Subsequent splicing defects primarily involve the use of cryptic 3′ splice sites that can induce mRNA degradation through nonsense-mediated decay or result in the production of dysfunctional proteins. Heterozygous *SF3B1* missense mutations, predominantly occurring within the HEAT repeats and including the K700E hotspot mutation, are frequently found in myelodysplasic syndromes with ring sideroblasts (MDS-RS) (*18*). Missplicing of the putative ISC transporter *ABCB7*, along with resultant protein down-regulation (*19*–*21*), has been reported in *SF3B1*-mutated hematologic pathologies and contributes to their pathogenesis (*22*). In this context, we describe how *SF3B1* mutations, leading to ABCB7 down-regulation, provide a genetic predisposition to cuproptosis. Overall, our work report advanced insights into copper ionophore activity in AML and its potential application for personalized therapy in *SF3B1*-mutated AMLs.

## RESULTS

### High-throughput screening in primary specimens identifies a molecule targeting *SF3B1*-mutated and poor-prognosis AMLs

**Fig. 1.**
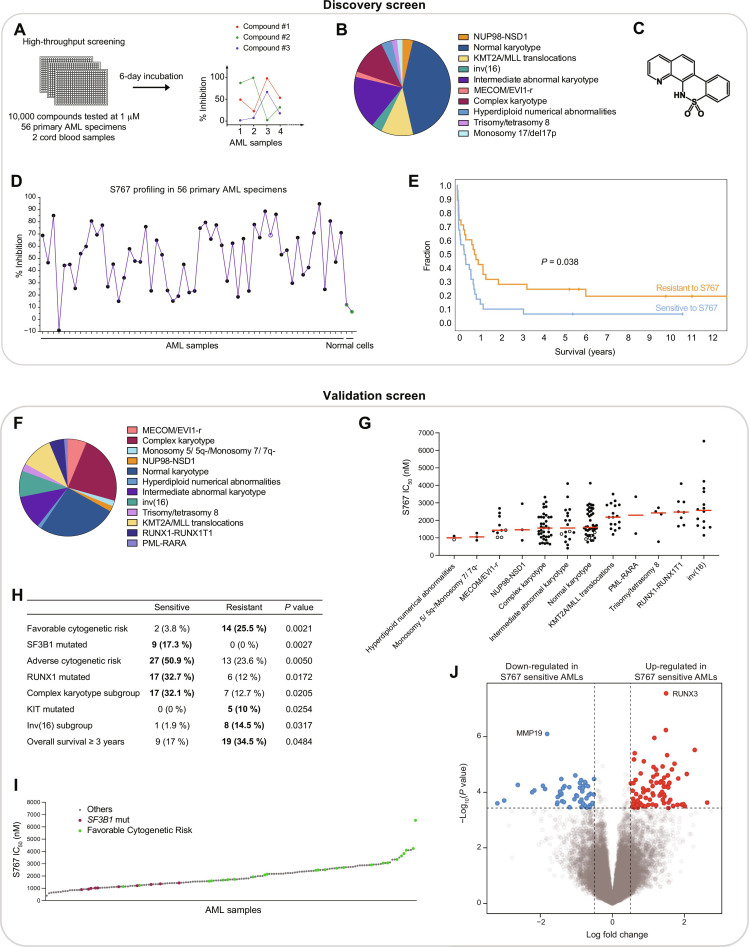
High-throughput screening in primary specimens identifies S767 targeting *SF3B1*-mutated and poor-prognosis AMLs. (**A**) High-throughput screening strategy. (**B**) AML subtype classification of the 56 primary specimens used in discovery screen. (**C**) Chemical structure of S767. (**D**) Inhibitory profile of S767 compound across 56 primary AML specimens (black dots) and 2 normal cord blood samples (green dots). Percentage of inhibition at 1 μM, normalized to dimethyl sulfoxide (DMSO) control treatment. Empty dot represents *SF3B1*-mutated sample. (**E**) Survival curve comparing the more sensitive (*n* = 28) versus the more resistant (*n* = 28) AML samples to S767, according to the median distribution. (**F**) AML subtype classification of the 161 primary AML specimens used in the validation screen. (**G**) Dot plot distribution of S767 IC_50_ values across 161 primary AMLs. The 10 samples carrying *SF3B1* mutation are displayed as empty dots. Median is represented in red. (**H**) Associations with S767 compound response were calculated by comparing the more sensitive (tier 1) and the more resistant (tier 3) AML specimens according to cytogenetic risks, AML subgroups, clinical data, and mutational status available for each variable tested. Only significant enrichments (*P* value < 0.05) are displayed. The number and percentage of samples present in sensitive versus resistant group is depicted. (**I**) Waterfall representation of S767 IC_50_ values obtained in the 161 primary AML specimens. (**J**) Volcano plot representation of differentially expressed genes in S767 sensitive (tier1) versus resistant (tier3) AMLs.

With the aim of identifying original molecules and therapeutic targets for AML, we performed a high-throughput screening assay of 10,000 compounds in 56 primary AML specimens and 2 normal cord blood specimens (Fig. 1A). The small molecule library of high structural diversity (Bristol Myers Squibb) included a majority of compounds with no known biological activity, as well as the typical clinical compounds now used to treat AML (e.g., venetoclax, daunorubicin, cytarabine, etc.). Biologically diverse primary AML specimens (Fig. 1B and fig. S1A) and normal CD34^+^ CB cells were treated for 6 days before assessing cell viability.

Profiles of inhibition across primary AMLs—or fingerprint—were then associated to each compound, looking for molecules with increased activity toward poor-prognosis AML samples and limited toxicity in normal cells (to exclude general cytotoxic compounds). At the tested dose of 1 μM, the S767 molecule (Fig. 1C) showed a unique and selective fingerprint in primary AMLs (>60% growth inhibition in 43% of the specimens, Fig. 1D and table S1), with low toxicity on normal CD34^+^ cord blood cells (median: 9.2% inhibition). Specimens sensitive to S767 had a poorer overall patient survival compared to resistant ones (Fig. 1E), while complex karyotype (CK) AMLs were among the most sensitive specimens (fig. S1B).

To better stratify S767 response in AML and possibly identify biomarkers, we tested S767 potency in dose-response assays and determined half-maximal inhibitory (IC_50_) concentration in a panel of 161 genetically diverse primary AMLs (Fig. 1, F and G; fig. S2A; and table S1). By comparing the most sensitive (tier 1) versus most resistant (tier 3) AML samples, we found that sensitive samples were enriched for *SF3B1*-mutated, adverse cytogenetic risk and CK AMLs (Fig. 1, H and I). On the other hand, more resistant AMLs associate with favorable cytogenetic risk and overall survival superior at 3 years.

Looking at differentially expressed genes in tier1 versus tier3 AML, we identified *RUNX3* as the most significant highly expressed gene in S767 sensitive samples (Fig. 1J and table S2). *RUNX3* expression anticorrelates with S767 IC_50_ values (fig. S2B) and stratifies S767 sensitivity within AML subgroups (fig. S2, C and D). High *RUNX3* expression is known to associate with poor cytogenetic risk AML subtypes and lower overall survival in AML (*23*), in line with S767 sensitivity. Gene set enrichment analysis also showed down-regulation of metabolic pathways in primary AML sensitive to S767 (fig. S2E).

Overall, we identified *SF3B1*-mutated and CK AMLs as particularly sensitive to S767 and *RUNX3* expression as a potential biomarker for S767 cytotoxic response.

### S767 exposure disrupts intracellular metal homeostasis

**Fig. 2.**
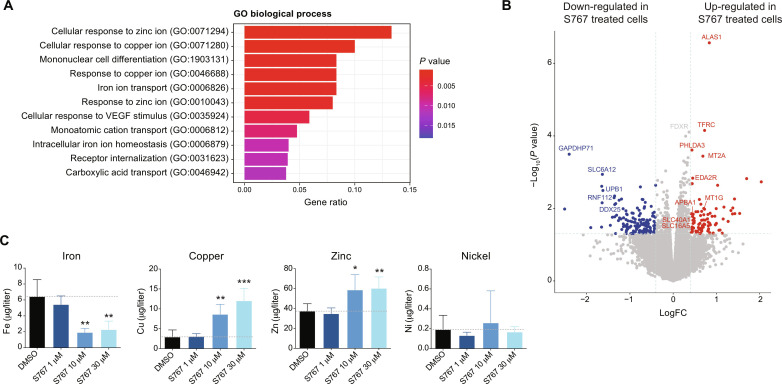
S767 exposure disrupts intracellular metal homeostasis. (**A**) Pathway analysis showing the biological processes significantly enriched in up-regulated genes [*P* value <0.05, log fold change (FC) > 0.4] following S767 treatment (1 μM, 24 hours), compared to DMSO. Top 11 gene ontology (GO) terms are shown in descending order. VEGF, vascular endothelial growth factor. (**B**) Volcano plot representing differentially expressed genes in S767 treated OCI-AML5 cells (1 μM, 24 hours) compared to DMSO. (**C**) Intracellular metal quantification by ICP-MS in OCI-AML5 cells exposed 3 hours to increasing concentration of S767. Data are represented as mean ± SD (*n* = 5, unpaired *t* test compared to DMSO condition).

To determine the mechanisms by which the S767 molecule mediates anti-AML activity, we performed a transcriptomic analysis of OCI-AML5 exposed 24 hours to 1 μM S767 (table S3). This revealed that biological processes linked to cellular response to metals, including zinc, copper, and iron, were up-regulated compared to dimethyl sulfoxide (DMSO)–treated cells (Fig. 2A). This also includes up-regulation of the *ALAS1* rate-limiting enzyme of heme biosynthesis, transferrin receptor *TFRC* and *MT2A* involved in metal detoxification (Fig. 2B). We then measured intracellular metal levels by inductively coupled plasma mass spectrometry (ICP-MS) in cells exposed to increasing concentration of S767 for 3 hours. We observed a significant decrease in intracellular iron, while copper and zinc levels were increased (Fig. 2C), indicating that S767 treatment induces a profound dysregulation of intracellular metal homeostasis.

### CRISPR-Cas9 screen identifies *ABCB7* as a sensitizing gene to S767-mediated cell death

**Fig. 3.**
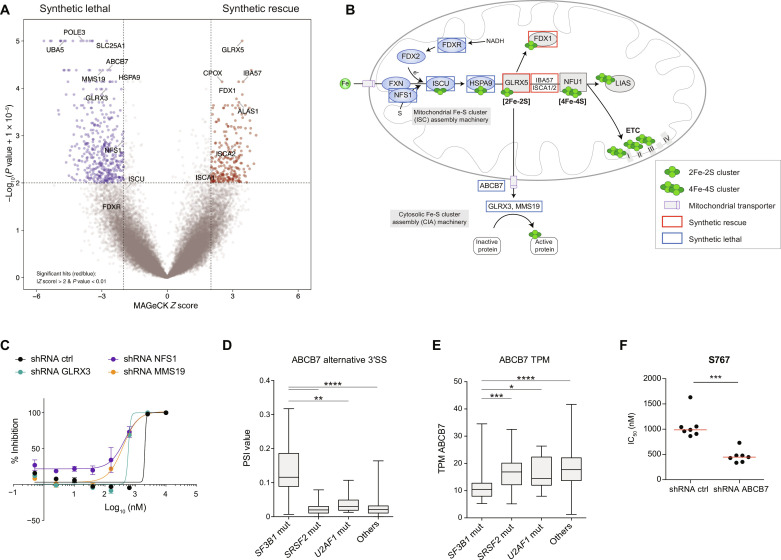
CRISPR-Cas9 screen identifies *ABCB7* as a sensitizing gene to S767-mediated cell death. (**A**) Volcano plot representing the results of whole-genome CRISPR-Cas9 loss-of-function screen performed in EKO OCI-AML5 cells upon exposure to the S767 compound (1.1 μM). (**B**) Simplified representation of the ISC biosynthetic pathways. Briefly, ISCs are assembled in the mitochondrial matrix by the ISC assembly machinery on the scaffold protein ISCU. Clusters are transferred to ISC trafficking proteins through HSPA9 to be loaded on targeted mitochondrial proteins or transferred to form 4Fe-4S clusters. NFU1 lastly transfers 4Fe-4S clusters to target proteins including ETCI-III and LIAS, among others. ISC are also exported to the cytoplasm, putatively through the ABCB7 transporter, and loaded to cytosolic and nuclear proteins by the CIA machinery, comprising GLRX3 and MMS19 proteins, among others. Top hits from the Fe-S cluster pathway identified as synthetic lethal and synthetic rescue genes in the CRISPR-Cas9 screen are framed in blue and red, respectively. (**C**) Representative dose-response curves for S767 obtained in OCI-AML5 cells constitutively expressing shRNAs targeting key genes of the ISC biogenesis (error bars indicate SD of technical duplicates). (**D**) Box plot representation of the percent-splice-in (PSI) value regarding *ABCB7* alternative 3′ splice site in primary AML specimens with or without mutations in the splicing factors *SF3B1*, *SRSF2*, or *U2AF1* (Mann-Whitney *U* test). (**E**) *ABCB7* mRNA expression expressed in TPM (transcripts per million) in primary AML samples carrying mutations in the indicated splicing factors (Mann-Whitney *U* test). (**F**) Dot plot distribution of S767 IC_50_ values in OCI-AML5 cells expressing an shRNA control or targeting *ABCB7*. Median is represented in red (*n* = 7, Mann-Whitney *U* test).

To determine synthetic lethal and rescue interactions with S767, we performed a genome-wide CRISPR-Cas9 loss-of-function screen (*24*) in which cells were exposed to 1.1 μM S767 compound for 10 doublings (Fig. 3A and table S4). CRISPR-Cas9 screen results pointed out mitochondrial metabolism as being critical for S767-mediated cytotoxic activity. In particular, loss of several genes involved in the biosynthesis or transport of ISC conferred synthetic lethality with S767 treatment (Fig. 3, A and B). Disrupting early steps in ISC biosynthesis through *FDXR*, *NFS1*, *ISCU*, and *HSPA9* or cytosolic Fe-S protein assembly machinery through *GLRX3*, *MMS19*, and *ABCB7* provides a synthetic lethal effect with S767 treatment. This observation was validated using short hairpin RNAs (shRNAs) targeting top hits *NFS1*, *GLRX3*, and *MMS19* (Fig. 3C). The opposite effect was observed when mitochondrial proteins *GLRX5*, *IBA57*, and *ISCA1/2*, in charge of loading Fe-S cofactor on mitochondrial proteins, were abrogated. These data suggest that ISC biogenesis is intimately linked to S767 cytotoxic response.

The synthetic lethal gene *ABCB7* is known to be aberrantly spliced and partly dysfunctional in *SF3B1*-mutated AMLs (*19*), which we identified as highly sensitive to S767 (Fig. 1, H and I). Analysis of the Leucegene transcriptomic data of primary AML specimens confirmed missplicing (Fig. 3D and table S5) and down-regulation of *ABCB7* mRNA expression (Fig. 3E) in *SF3B1*, but not *SRSF2* and *U2AF1*, mutated AMLs (table S6). We further validated that *ABCB7* down-regulation by shRNA sensitizes cells to S767 (Fig. 3F), suggesting a molecular explanation to *SF3B1*-mutated AML sensitivity toward S767.

### Identification of UM4118 as a potent and selective analog of S767

**Fig. 4.**
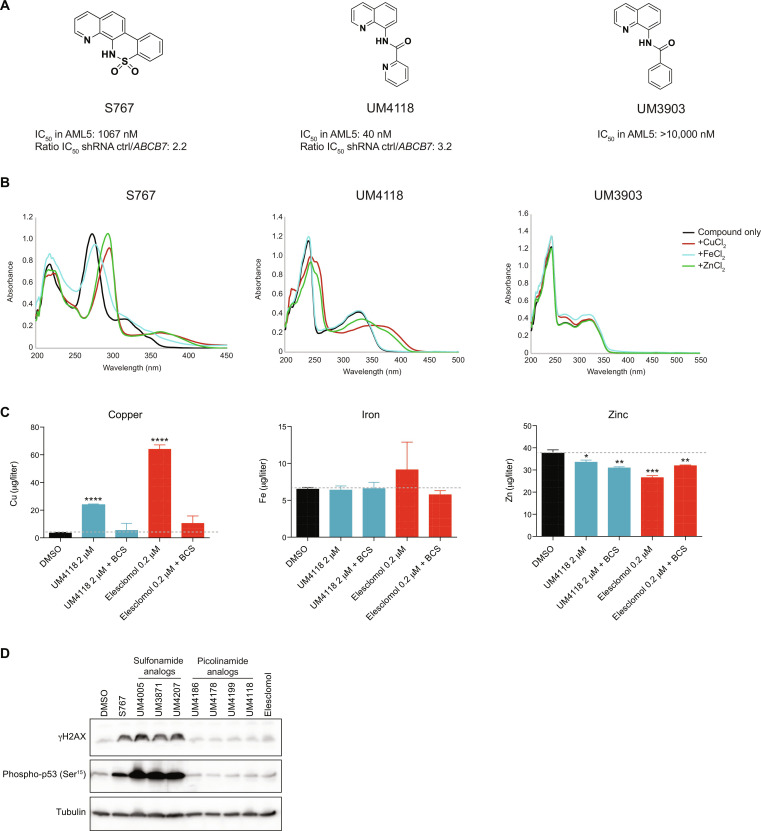
Identification of UM4118 as a potent and selective analog of S767. (**A**) Structures of S767 [C7-locked *N*-(quinoline-8-yl)benzenesulfonamide], UM4118 [*N*-(quinoline-8-yl)picolinamide], and UM3903 [*N*-(quinoline-8-yl)benzamide] with IC_50_ values in OCI-AML5 cells. Ratio of IC_50_ values obtained in OCI-AML5 stably expressing shRNA control over shRNA *ABCB7* is indicated. (**B**) Ultraviolet-visible spectrum of S767, UM4118 and UM3903 (40 μM) alone and in the presence of indicated metals (40 μM) in ethanol at room temperature. (**C**) Intracellular metal quantification by ICP-MS in OCI-AML5 cells exposed 3 hours to indicated compounds with and without 100 μM the non-cell permeable copper chelator BCS. Data are represented as mean ± SD (*n* = 3, unpaired *t* test compared to DMSO condition). (**D**) Immunoblot of γH2A.X and phospho-p53 at serine 15 from OCI-AML5 cells treated 24 hours with S767 analogs or elesclomol. For each compound, a concentration corresponding to 12 times the IC_50_ was used. Tubulin is used as a loading control.

Because S767, a C7-locked *N*-(quinoline-8-yl)benzenesulfonamide, has low potency (IC_50_ values in the micromolar range) in both cell lines and primary specimens, in addition to a poorly functionalizable scaffold and a wide effect on intracellular metal homeostasis, we decided to optimize S767 potency and selectivity through extensive structure-activity relationship (SAR) studies. On the basis of the sensitizing effect observed while *ABCB7* transporter is depleted, we have synthetized and tested approximately 100 analogs, focusing on molecules for which IC_50_ values would be at least two times lower in *ABCB7* down-regulated cells compared to the control cell line (representative examples in fig. S3A). This SAR revealed the UM4118 molecule, as part of an “open-form” series of analogs, which achieved potency in the nanomolar range, further potentiated in *ABCB7* depleted cells (Fig. 4A). As observed for S767, UM4118 is able to complex metals, in particular copper and zinc (Fig. 4B, see shift in absorbance curves), while analogs with structural modifications that abrogated metal binding were inactive in cells [see UM3903 in Fig. 4 (A and B) and fig. S3B for additional examples]. This indicates that metal binding is essential for compound activity and that perturbations of intracellular metal homeostasis might be a direct effect of these metal-binding molecules.

Notably, ICP-MS analysis of cells exposed 3 hours to UM4118 showed that this optimized analog specifically increases intracellular copper levels, while iron and zinc levels were poorly affected (Fig. 4C). Extracellular copper chelation using bathocuproine disulfonic acid (BCS) counteracts the increase of intracellular copper levels after UM4118 exposure, suggesting that the molecule import copper from media into the cell. Moreover, data obtained with UM4118 compare to the known copper-ionophore elesclomol (*25*, *26*), suggesting that UM4118 activity has been optimized toward copper interaction. Because copper complexes can damage DNA (*27*–*29*), we monitored DNA lesions following 24-hour exposure to representative molecules and showed that S767 as well as its sulfonamide class of analogues induced large amounts of γH2AX, in contrast to UM4118 and the picolinamide series (Fig. 4D and fig. S3A). Together, SAR led to the identification of the metal binding UM4118 molecule as a more potent (26-fold increase in IC_50_ in OCI-AML5), copper-selective, and nongenotoxic analog of S767 for further characterization.

### UM4118 acts as a copper ionophore which cytotoxicity relies on mitochondrial respiration

**Fig. 5.**
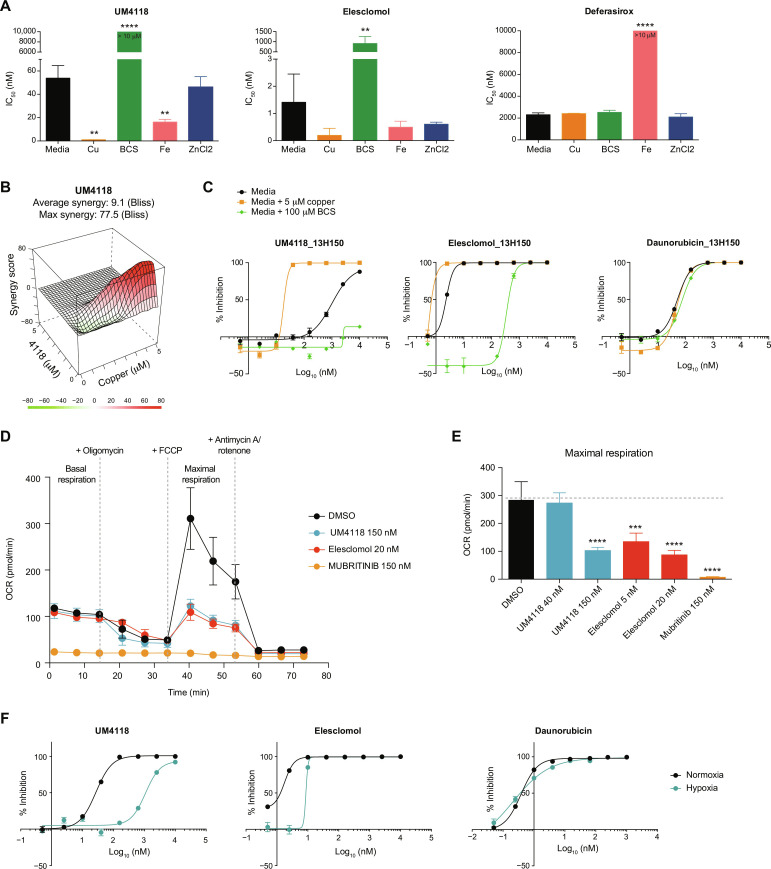
UM4118 acts as a copper ionophore. (**A**) IC_50_ values of UM4118, elesclomol, and deferasirox (iron chelator used as control) determined in regular media, in media supplemented with 5 μM copper (Cu), 10 μM iron (Fe), 10 μM zinc (ZnCl_2_) or in the presence of the copper chelator BCS (100 μM) in OCI-AML5 cells. Data are represented as mean ± SD (*n* = 3, unpaired *t* test compared to media condition). (**B**) Synergistic interaction between UM4118 and copper in OCI-AML5 cells calculated with the Bliss method. (**C**) Dose-response curves obtained in CK primary AML sample 13H150 exposed to indicated compounds in regular AML media, supplemented with 5 μM copper or 100 μM BCS (error bars indicate SD of technical duplicates). Daunorubicin is used as a negative control. (**D**) Effect of a 24-hour exposure to DMSO, elesclomol, UM4118, and mubritinib (positive control, ETC inhibitor) on oxygen consumption rates (OCR) in 100,000 OCI-AML5 cells determined by Seahorse assay (mean ± SD, *n* = 6). (**E**) Quantification of the effect of compounds on maximal respiration as measured in (D). Data are represented as mean ± SD (*n* = 6, unpaired *t* test compared to DMSO condition). (**F**) Dose-response curves of OCI-AML5 cells grown in normoxic (21% O_2_) or hypoxic (1% O_2_) conditions and exposed to indicated compounds (error bars indicate SD of technical duplicates). Daunorubicin is used as a negative control.

We hypothesized that, similar to elesclomol, UM4118’s cellular activity relies on its ability to bring copper into the cell and acts essentially as a copper ionophore. Accordingly, cellular activity of UM4118 is highly dependent on copper, as its supplementation in the culture media strongly enhances the cytotoxicity of this molecule, while it is abrogated when extracellular copper chelation is used (Fig. 5A). As a control, copper supplementation did not affect cytotoxicity of deferasirox, a known iron chelator, while iron supplementation rescued these cells. In addition, strong synergistic interaction was observed between UM4118 and copper at low concentrations (Fig. 5B). In primary AML specimens which are cultured in fetal bovine serum (FBS)–free media, thus removing the extracellular source of copper, we also observed that UM4118 cytotoxic activity is highly dependent on the presence of extracellular copper (Fig. 5C and fig. S4A). Again, the copper ionophore elesclomol showed similar results to UM4118 (Fig. 5, A and C, and fig. S4A). Of note, down-regulation of the unique copper importer *CTR1* (*SLC31A1*) did not affect UM4118 and elesclomol mediated toxicity (fig. S4B), indicating that copper does not shuttle through regulated copper import systems after exposure to UM4118 but rather support the conclusion that UM4118 acts as a copper ionophore.

As a consequence of copper overload, elesclomol impairs mitochondrial respiration indirectly through the inhibition of components of the TCA cycle (*8*). Accordingly, we observed that UM4118 and elesclomol impair maximal mitochondrial respiration capacity (Fig. 5, D and E) without affecting basal respiration rates as would do a direct ETC1 inhibitor [see mubritinib in Fig. 5 (D and E)]. In addition, cells that were grown under hypoxic conditions (1% O_2_) were more resistant to UM4118 (Fig. 5F), highlighting the fact that copper ionophore–mediated cell death implies mitochondrial respiration function.

### UM4118 induces cell death by cuproptosis

**Fig. 6.**
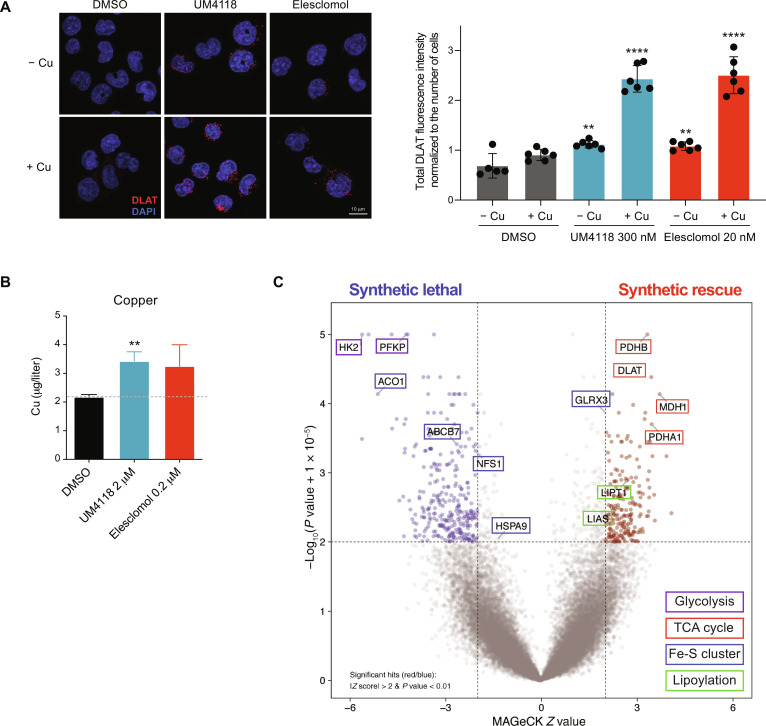
UM4118 induces cell death by cuproptosis. (**A**) Representative images (left) of DLAT staining (red) in OCI-AML5 exposed 16 hours to 300 nM UM4118 or 20 nM elesclomol in media ± 1 μM copper. Nuclei are counterstained with 4′,6-diamidino-2-phenylindole (DAPI; blue). Quantification (right) of total DLAT signal divided per the number of cells in each image is depicted (unpaired *t* test compared to DMSO condition). (**B**) Intramitochondrial copper quantification by ICP-MS in human embryonic kidney 293 cells exposed 3 hours to indicated compounds. Data are represented as mean ± SD (*n* = 3, unpaired *t* test compared to DMSO condition). (**C**) Volcano plot representing the results of whole-genome CRISPR-Cas9 loss-of-function screen performed in EKO OCI-AML5 cells upon exposure to UM4118 (110 nM).

To confirm UM4118 mechanism of action as a copper ionophore and that cuproptosis was causing cell death, we looked at DLAT protein aggregation that occurs through the binding of excess copper to lipoylated DLAT and leads to proteotoxic stress (*8*). We indeed observed the presence of DLAT aggregates by immunofluorescence upon UM4118 and elesclomol treatment, which is exacerbated in the presence of copper supplementation (Fig. 6A). We also confirmed that UM4118 increases intramitochondrial copper levels by ICP-MS analysis on mitochondria extracts (Fig. 6B and fig. S5A). Of note, pretreatment with ferroptosis inhibitor (Ferrostatin-1), caspase 3 inhibitor (Z-DEVD-FMK), or pan caspase inhibitor (Emricasan) failed to rescue UM4118 cytotoxic effect (fig. S5B), confirming that ferroptosis or apoptosis is not involved in UM4118 cytotoxic activity. Last, we conducted a genome-wide CRISPR-Cas9 loss-of-function screen with UM4118 molecule to identify synthetic interactions (Fig. 6C and table S7). Results first revealed that, knockdown of *HK2* or *PFKP*, two enzymes involved in glycolysis, showed strong synthetic lethality, supporting UM4118 mechanism of action through inhibition of cellular respiration (Fig. 5, D to F). Notably, genetic suppression of the pyruvate dehydrogenase complex (PDC) by targeting *DLAT*, *PDHB*, and *PDHA1* rescued cells from UM4118 treatment. These results are in line with Tsvetkov *et al.* (*8*) showing that disruption of PDC rescues ovarian cells from elesclomol-mediated cuproptosis. Accordingly, disruption of *DLAT* or key enzymes mediating its lipoylation (*LIAS* and *LIPT1*) provides synthetic rescue over UM4118 treatment (Fig. 6C). However, validation experiment using shRNA targeting *DLAT* showed a partial rescue of UM4118-mediated cell death, suggesting that DLAT participates but does not recapitulate all the cellular effects of UM4118 in AML cells (fig. S5, C and D). Together, these data support the hypothesis that UM4118 mediates cuproptosis.

### ISC deficiency potentiates copper-mediated cell death

**Fig. 7.**
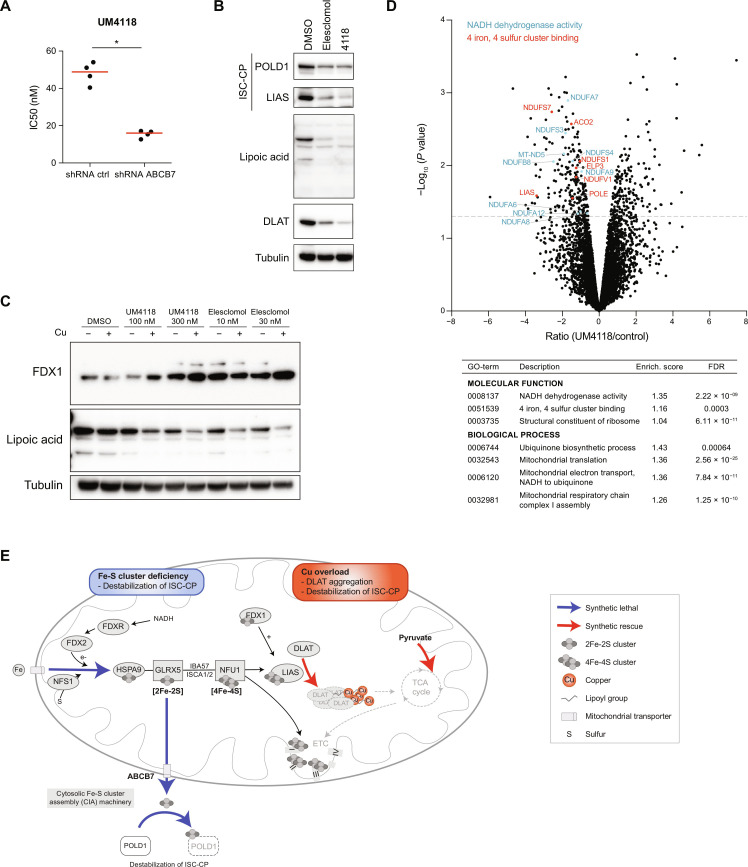
ISC deficiency potentiates copper-mediated cell death. (**A**) Dot plot distribution of UM4118 IC_50_ values in OCI-AML5 cells expressing an shRNA control or targeting *ABCB7*. Median is represented in red (*n* = 4, Mann-Whitney *U* test). (**B**) Immunoblot analysis of POLD1, LIAS, lipoic acid, and DLAT proteins on OCI-AML5 cells cultured in media supplemented with 1 μM copper and exposed 16 hours to 30 nM elesclomol, 300 nM UM4118, or DMSO. Tubulin is used as a loading control. (**C**) Immunoblot analysis of FDX1 and lipoic acid proteins on OCI-AML5 cells treated with indicated compounds for 16 hours, both with and without media supplementation with 1 μM copper. Tubulin is used as a loading control. (**D**) Total proteome analysis performed on OCI-AML5 cells exposed 16 hours to UM4118 (300 nM) in media supplemented with 1 μM copper. Data represent proteins identified in at least two peptides. Some of the most significant GO terms (STRING) are indicated in the table. Significant proteins of the “NADH dehydrogenase activity” and “4 iron, 4 sulfur cluster binding” pathways are highlighted (NDUFS7, NDUFS1, and NDUFV1 belong to both pathways). (**E**) In this simplified model, copper overload leads to the aggregation of lipoylated DLAT and destabilization of ISC-containing proteins (ISC-CP). LIAS, which mediates DLAT lipoylation, requires an ISC for its activity and is depleted upon exposure to copper ionophores. ISC deficiency caused by the depletion of key genes of ISC biogenesis causes an additional destabilization of ISC-containing proteins leading to synthetic lethality with copper overload. Key processes identified in the UM4118 CRISPR experiment showing synthetic lethality or synthetic rescue with UM4118 are represented by blue and red arrows, respectively.

As observed with S767 (Fig. 3, A and B), disruption of ISC biosynthesis (*NFS1* and *HSPA9*), as well as loss of the *ABCB7* transporter, causes synthetic lethality with UM4118 (Fig. 6C). We confirmed that down-regulation of *NFS1* and *NFU1*, as well as *ABCB7*, indeed increased sensitivity to UM4118 in OCI-AML5 cells (Fig. 7A and fig. S6A). A possible explanation to the synthetic lethality between copper overload and ISC deficiency is that copper excess causes a depletion of ISC containing proteins, through an unknown mechanism (*8*). We indeed observed that short time exposure to UM4118 depletes the ISC proteins POLD1 and LIAS (Fig. 7B). As a consequence of LIAS reduction, total lipoic acid proteins are depleted, including DLAT, indicating that copper overload impairs the lipoylation process ultimately. DLAT aggregation observed previously (Fig. 6A) also likely contributes to the depletion of soluble DLAT. As recently reported, FDX1 regulates lipoylation (*30*, *31*), and we observed an increase of FDX1 protein levels upon UM4118 and elesclomol exposure (Fig. 7C), which could be a compensatory effect to the depletion of LIAS and/or lipoic acid proteins.

Total proteome analysis revealed a significant depletion of mitochondrial proteins involved in electron transfer along the respiratory chain after exposure to UM4118 (Fig. 7D and table S8), which could contribute to the observed deficiency in mitochondrial respiration. Depletion of 4Fe-4S cluster containing proteins, including LIAS, was also highly significant. We hypothesized that by disrupting ISC homeostasis, an additional layer of ISC protein deficiency adds up to the effect of copper overload, explaining the observed synthetic lethality with UM4118 (see proposed model in Fig. 7E).

### * SF3B1* mutations, through *ABCB7* deficiency, sensitize AML cells to copper ionophores

**Fig. 8.**
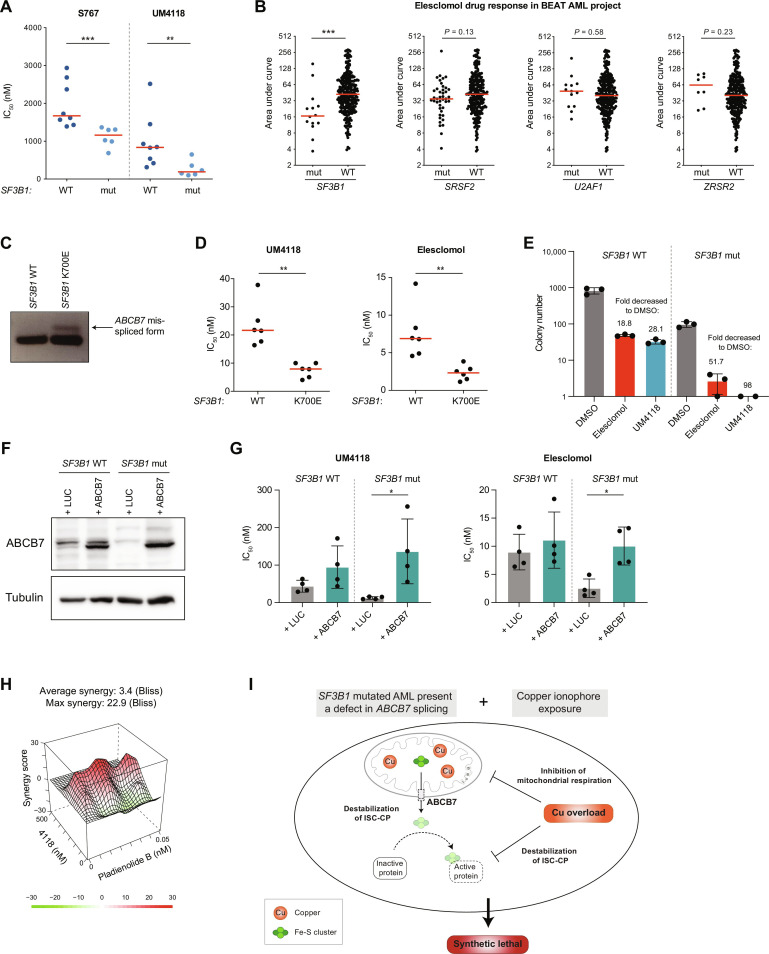
*SF3B1* mutations, through *ABCB7* deficiency, sensitize AML cells to copper ionophores. (**A**) Dot plot distribution of S767 and UM4118 IC_50_ values across 14 primary AML specimens carrying or not *SF3B1* mutation. Median is represented in red, Mann-Whitney *U* test. (**B**) Dot plot distribution of response to elesclomol treatment in the BEAT AML cohort (default settings). Area under curve retrieved from Vizome (BEAT AML data viewer). Median is represented in red, Mann-Whitney *U* test. (**C**) Reverse transcription PCR products of *ABCB7* mRNA amplification obtained in K562 isogenic cell lines for *SF3B1* (WT or carrying the K700E heterozygous mutation). (**D**) Dot plot distribution of UM4118 and elesclomol IC_50_ values in K562 *SF3B1* WT or mutated cell lines (*n* = 6, Mann-Whitney *U* test). Median is represented in red. (**E**) The number of colonies coming from K562 cell lines carrying the *SF3B1* K700E heterozygous mutation or not was assessed in methylcellulose after 10 days. Cells were treated 48 hours with DMSO or 20 nM of the copper ionophores before seeding in methylcellulose. Mean fold decreased in colony numbers compared to DMSO is depicted. (**F**) Immunoblot analysis of ABCB7 protein in K562 WT or *SF3B1*-mutated cells infected with a lentiviral vector expressing either *ABCB7* cDNA or luciferase (LUC) as a control. Tubulin is used as a loading control. (**G**) Dot plot distribution of UM4118 and elesclomol IC_50_ values in K562 WT or *SF3B1*-mutated cells infected with a lentiviral vector expressing *ABCB7* or luciferase. Data are represented as the mean ± SD (*n* = 4, Mann-Whitney *U* test). (**H**) Synergistic interaction between UM4118 and pladienolide B in OCI-AML5 cells calculated with the Bliss method. (**I**) The proposed model implies that missplicing of *ABCB7* in *SF3B1*-mutated cells destabilizes the ISC-containing proteins (ISC-CP) which are further affected by copper overload, leading to a synthetic lethal interaction.

As we identified *SF3B1*-mutated AML as highly responsive to S767 (Fig. 1, H and I) and optimized UM4118 activity on *ABCB7* depleted cells (Fig. 4A), we tested UM4118 potency in a panel of 14 primary AMLs carrying or not *SF3B1* mutations. We observed that *SF3B1*-mutated specimens—coming from different AML subgroups—were significantly more sensitive to UM4118 (Fig. 8A). Although tested on a small cohort of primary AMLs, anticorrelation between high *RUNX3* levels and UM4118 sensitivity was observed, as previously found with S767 (fig. S7A). Extending our observations to the BEAT AML cohort (*32*), reporting the activity of elesclomol on 299 AML samples, we confirmed that *SF3B1*-mutated AMLs are significantly more sensitive to elesclomol than *SF3B1* WT AMLs (Fig. 8B and fig. S7B). This is not observed on samples bearing mutations on the other splicing factors *SRSF2*, *U2AF1*, and *ZRSR2*, highlighting the specific association with *SF3B1*.

Using an isogenic K562 cell line carrying the recurrent K700E *SF3B1* mutation, we confirmed the presence of a misspliced form of *ABCB7* mRNA (Fig. 8C) which is absent in their wild-type (WT) counterparts. While UM4118 exposure triggers cuproptosis in both K562 cell lines (fig. S7, C to E), *SF3B1*-mutated cells showed an increased sensitivity toward UM4118 and elesclomol (Fig. 8D) and decreased clonogenic potential after short time exposure to both copper ionophores (Fig. 8E), compared to WT K562 cells. We then overexpressed ABCB7 in *SF3B1*-mutated K562 cells, to restore ABCB7 protein levels (Fig. 8F), and showed that it partially rescues UM4118 and elesclomol cytotoxicity (Fig. 8G), suggesting a critical role of ABCB7 in *SF3B1*-related sensitivity.

Pladienolide B, a splicing inhibitor that binds to SF3B1, also showed a strong synergy with UM4118 at low doses (Fig. 8H), providing additional evidence of the interaction between SF3B1 function and copper ionophore sensitivity. Together, these results demonstrate that *SF3B1* alterations associate with an increased sensitivity to copper ionophores in AML which is mediated, at least in part, through *ABCB7* defects (see proposed model in Fig. 8I).

## DISCUSSION

Using a phenotypical screen on primary specimens of diverse genetic make-up, we identified S767 as a selective molecule in AML. Through compound optimization, we developed a more potent, specific, and nongenotoxic analog of S767, namely, UM4118, which acts as a copper ionophore. Our results further highlight previously unidentified mechanistic connections between ISC and copper-related cell death that can be leveraged to sensitize cells to cuproptosis. We found that ISC deficiency is synthetically lethal to copper-overload strategies in AML. Pragmatically, this is exemplified by the high sensitivity of *SF3B1*-mutated AMLs, in which *ABCB7* is misspliced and down-regulated. In addition, ABCB7 overexpression partially rescued UM4118-induced cell death in *SF3B1*-mutated cells, suggesting that ABCB7 plays a crucial role in sensitizing *SF3B1*-mutated AMLs to cuproptosis.

One possible explanation for the synthetic lethality observed between copper overload and ISC deficiency is that copper excess causes a depletion of ISC-containing proteins. However, how copper affects ISC-containing proteins stability and whether the latter contributes to cuproptosis remains unclear. In bacteria, it is known that copper can displace iron form ISC-containing proteins and account for copper toxicity (*33*–*35*). In addition, excess copper has been shown to block mitochondrial ISC protein maturation by inhibiting the ISC assembly machinery (*34*). These findings may explain the reported destabilization of ISC-containing proteins upon copper overload. Accordingly, we found that genetic suppression of the ISC pathway (i.e., knockdown of *NFS1*, *HSPA9*, or *ABCB7*) is synthetically lethal with S767 or UM4118 treatments, as it would amplify the ISC deficiency provoked by copper overload. These two processes are intimately linked as both ISC deficiency and copper excess impair mitochondrial respiration. ISCs are essential cofactors of the respiratory chain complexes I to III, while copper leads to TCA cycle impairment. LIAS, which mediates DLAT lipoylation, is itself an ISC-containing protein destabilized by copper overload. Data from the literature, in addition to our work, suggest that ISC deficiency and cuproptosis could interconnect at multiple levels.

*SF3B1* mutations lead to the aberrant splicing of hundreds of mRNAs in AML. We identified 391 aberrant splicing events associated to *SF3B1* mutation in the Leucegene cohort, which are enriched (43.7%) for alternative 3′ splicing defects (table S5 and fig. S8A). While we demonstrated that *ABCB7* missplicing sensitizes *SF3B1* mutated cells to cuproptosis, others may also contribute to this effect. mRNAs of the ISC containing proteins *NDUFS7*, *CIAPIN1*, and *POLE* were found with significant splicing alterations in AML, as well as *UQCC1*, *COX18*, and *DLST*, involved in mitochondrial respiration (fig. S8B). Although the total mRNA expression of these genes was not affected by the missplicing events, functional consequences of these events are not known. Recently, *SF3B1* mutations in breast and uveal cancers were associated to metabolic dysregulation, characterized by a decrease in OXPHOS (*36*, *37*). Whether missplicing of metabolic genes leads to a defect in mitochondrial respiration in AML is an interesting area of investigation as this could represent another level of sensitization toward copper ionophores. In line with this hypothesis, we observed that primary AML samples showing the highest sensitivity toward S767 share down-regulated metabolic transcriptional signatures (figs. S2E and S8C), which could have predisposed cells to S767-associated cytotoxicity.

Despite the strong in vitro antineoplastic activities of copper ionophores, as well as their selectivity toward tumor cells (*25*, *38*), clinical trials using the copper ionophores elesclomol and disulfiram failed to produce significant benefits for cancer treatment (*39*–*45*). Drug delivery and limited bioavailability, along with the lack of robust biomarkers in these untargeted clinical trials, might be responsible for this relative failure. In the meantime, our work adds up UM4118 molecule at the known repertoire of copper ionophore molecules for future therapeutic development. UM4118 is structurally distinct from elesclomol and disulfiram and could offer alternative pharmacodynamic properties to successfully translate into in vivo applications.

Our work highlights that *SF3B1* mutations, recognized in the 2022 ELN risk classification (*46*) as an adverse prognostic marker if they are associated with intermediate or adverse risk AML, provide a genetic predisposition to copper overload–based therapies, offering a unique therapeutic angle for these patients. In addition, our results suggest that adverse cytogenetic risk AMLs, including CK, with the use of *RUNX3* expression levels as a biomarker, might be another group of AML patients to target. *SF3B1* mutations are also found in 20% of MDS cases, in which a strong enrichment is found in MDS with ring sideroblasts (*18*), 15% of chronic lymphocytic leukemias (*47*) and 6% of chronic myelomonocytic leukemia (*48*). In addition, *SF3B1* somatic mutations are present in a range of solid tumors, with high frequency (around 20%) in uveal melanoma (*49*, *50*) and more rarely in cases of breast cancers (*51*) or cutaneous melanomas (*52*) for example. Whether *SF3B1* mutations would confer sensitivity to copper ionophores in other hematologic neoplasms and solid tumors is of great interest and requires further evaluation.

Together, we describe how a nonprotein targeting molecule exploits a genetic vulnerability associated to ISC deficiency in AML. Last, we propose *SF3B1* mutations as a biomarker and mechanism of action for copper ionophore–directed therapies to be used in future clinical trials.

## MATERIALS AND METHODS

### Study approval

The Leucegene project is an initiative approved by the Research Ethics Boards of Université de Montréal and Maisonneuve-Rosemont Hospital. All leukemia samples and paired normal DNA specimens were collected and characterized by the Quebec Leukemia Cell Bank after obtaining an institutional Research Ethics Board–approved protocol with informed consent according to the Declaration of Helsinki. The Quebec Leukemia Cell Bank, a biobank certified by the Canadian Tissue Repository Network, is a research axis of The Cancer Research Network (RRCancer), a thematic network of the Fonds de recherche du Québec–Santé. Material and data were transferred to the principal investigator after signing a material transfer agreement adapted from the documents prepared by the RRCancer (https://rrcancer.ca/en/biobanking-documents/).

### Primary AML sample culture and chemical screens

Freshly thawed primary AML specimens were used for chemical screens following the procedure previously described in (*53*). Compounds were dissolved in DMSO, diluted in media immediately before use, and added to seeded cells at the unique concentration of 1 μM for the primary screen or in serial dilution (8 dilutions, 1:3, 10 μM down to 4.5 nM) for the validation screen, in duplicate wells.

### Whole-genome CRISPR-Cas9 deletion screens

Following procedure previously described in (*53*), we used the extended knockout (EKO) pooled lentiviral library (*24*) to perform whole-genome CRISPR-Cas9 loss-of-function screen. Briefly, OCI-AML5 EKO cells were cultured in 10% FBS Dulbecco’s modified Eagle’s medium supplemented with doxycycline (2 μg/ml) for a period of 7 days to induce knockouts. The knockout library was maintained in culture 14 more days with exposure to 1.1 μM S767, 110 nM UM4118, or DMSO (without doxycycline).

### Compounds

The following compounds were used for cell treatment: deferasirox (Adooq, A10293), elesclomol (eNovation Chemicals), ferrostatin-1 (Sigma-Aldrich, SML0583), emricasan (Cayman Chemical, 22204), Z-DEVD-FMK (Cayman Chemical, 14414), pladienolide B (Santa Cruz, sc-391691), Mubritinib (Selleckchem, S2216), and BCS (Santa Cruz, sc-217698).

### Inductively coupled plasma mass spectrometry

OCI-AML5 cells to be analyzed were treated with the indicated compounds for 3 hours in media supplemented by 1 μM copper. Treatment was done at a density of 1 million cells/ml, and 10 million cells were then washed twice in phosphate-buffered saline (PBS) and digested overnight in 100 μl of Suprapur 65% nitric acid (Millipore Sigma, 1004410250) at room temperature. For intramitochondrial quantification, 2 × 10^7^ human embryonic kidney 293 cells were used per replicate. The mitochondria were isolated on the basis of the scaled down (20×) procedure described in (*54*). Crude mitochondrial pellets were resuspended and digested overnight in 100 μl of Suprapur 65% nitric acid at room temperature. The samples were then diluted 50× in high-performance liquid chromatography–grade water (Thermo Fisher Scientific, W5-4) and analyzed. Blank samples were spiked with reference metal standards and used for quantification. The quantification of copper, zinc, iron, and nickel by ICP-MS was performed by the CACEN platform at Université de Montréal on a NexION 5000 apparatus (PerkinElmer).

### RNA sequencing and splicing analysis in primary AML samples

RNA sequencing (RNA-seq) libraries were constructed according to TruSeq Protocols (Illumina), and sequencing was performed using an Illumina HiSeq 4000 instrument. Trimming of sequencing adapters and low-quality bases was done using the Trimmomatic (v0.38) tool (*55*). Resulting reads were mapped to the reference using the RNA-seq aligner STAR (v2.7.1) (*56*), and quantification of gene and isoform expression were performed using RSEM (v1.3.2) (*57*). Differential expression analyses were performed using the Limma-Voom R package (*58*). Alternative splicing features, PSI/Ψ (Percent Spliced-In) values obtained for exon skipping, alternative 5′ or 3′ splice sites, mutually exclusive exons, and retained introns were evaluated directly from unclipped aligned reads using the rMATS algorithm (v4.0.1) (*59*).

### Transcriptome analysis of OCI-AML5 cells treated with S767

OCI-AML5 cells were treated in triplicates with 1 μM S767 for 24 hours. Total RNA was isolated using TRIzol as recommended by the manufacturer (Invitrogen) and purified using RNeasy Micro columns (Qiagen). RNA-seq libraries were constructed using the New England Biolabs (NEB) mRNA stranded kit, and sequencing was performed using an Illumina NovaSeq 6000 instrument. Analysis was performed as detailed above. The RNA-seq data are accessible at Gene Expression Omnibus (GEO; GSE241922).

### Reverse transcription PCR validation of ABCB7 missplicing

RNA from K562 cell lines was harvested in TRIzol (Thermo Fisher Scientific) and isolated according to the manufacturer’s protocol and reverse-transcribed using Moloney Murine Leukemia Virus (MMLV) reverse transcriptase and random primers (Thermo Fisher Scientific). *ABCB7* amplification by polymerase chain reaction (PCR; 40 cycles) was performed using the following primers: 5′-ATGATGCAGGTAATGCTGCT-3′ and 5′-TCAGCATAGCCAGAGTAGAGGT-3′ and allowed amplification of a 153 bp (canonical form) and 174 bp (misspliced form) product (*19*). PCR products were run on 4% agarose gel and visualized using an ultraviolet transilluminator.

### Ectopic expression of ABCB7

Two gene fragments containing overlapping sequences of the *ABCB7* coding sequence were synthesized by Twist Biosciences and cloned into the MNDU-pgk-GFP lentiviral vector by Gibson assembly. Cells were infected with lentivirus in media supplemented with polybrene (10 ng/ml) for 48 hours. Cells were then washed in PBS, counted, and plated for dose-response assays.

### Statistical analyses

Statistical testing was performed by using the MAGeCK-VISPR-MLE method (*60*) for CRISPR screens, the Limma-Voom method (*58*) for RNA-seq, or GraphPad Prism version 6.0. for statistical functions (Mann-Whitney test or unpaired *t* test as indicated in the figure legends). Results having *P* value < 0.05 were considered significant. Statistical differences of *P* < 0.05, *P* < 0.005, *P* < 0.0005, and *P* < 0.0001 are depicted as *, **, ***, and **** respectively in figures. Additional methods are available in the Supplementary Materials.

## Supplementary Materials

### This PDF file includes:

Supplementary Materials and Methods Figs. S1 to S8 Legends for tables S1 to S8 References
