# Supplementary material for: SF3B1 mutations provide genetic vulnerability to copper ionophores in human acute myeloid leukemia
Source: Sci Adv. 2024 Mar 22;10(12):eadl4018. doi: 10.1126/sciadv.adl4018 (PMC10959413; doi:10.1126/sciadv.adl4018)
Supplement: Supplementary file 1 — Supplementary Materials and Methods Figs. S1 to S8 Legends for tables S1 to S8 References [file sciadv.adl4018_sm.pdf]

Science Advances
AAAS

Supplementary Materials for

SF3B1 mutations provide genetic vulnerability to copper ionophores in human acute myeloid leukemia

Céline Moison *et al.*

Corresponding author: Céline Moison, celine.moison@umontreal.ca; Anne Marinier, anne.marinier@umontreal.ca; Guy Sauvageau, guy.sauvageau@umontreal.ca

Sci. Adv. **10**, eadl4018 (2024)
DOI: 10.1126/sciadv.adl4018

The PDF file includes:

Supplementary Materials and Methods
Figs. S1 to S8
Legends for tables S1 to S8
References

Other Supplementary Material for this manuscript includes the following:

Tables S1 to S8

Materials and Methods

Cytogenetic analyses and cohort definitions

Cytogenetic aberrations and composite karyotypes of the Leucegene cohort were described according to the International System for Human Cytogenomic Nomenclature 2016 guidelines. Complex karyotype was defined as having 3 or more clonal chromosomal abnormalities in the absence of the recurrent genetic abnormalities, including t(8;21), inv(16) or t(16;16), t(9;11), t(6;9), inv(3) or t(3;3) and AML with *BCR-ABL1* (*61*).

Human cord blood cell collection and processing

Fresh umbilical cord blood units were collected from consenting donors according to ethically approved procedures at St Justine, Maisonneuve-Rosemont (Montréal, QC, Canada) and Charles Le Moyne (Longueuil, QC, Canada) Hospitals. Human $CD34^+$ cells were isolated using the EasySep Human Cord Blood $CD34^+$ Selection Kit (StemCell Technologies Cat #18056) through positive magnetic selection. Chemical screen was performed as described in primary AML with the following modifications: 2000 cord blood cells were seeded, CB media contains StemSpan-ACF (StemCell Technology #9855) supplemented with 100 ng/mL stem cell factor (Shenandoah #100-04), 100 ng/mL FLT3L (Shenandoah #100-21), 50 ng/mL TPO (R&D #288-TP-200/CF), 10ug/mL Human LDL (StemCell Technology #026989), 1X glutamax (Invitrogen #35050061), gentamicin (50 μg/mL), ciprofloxacin (10 μg/mL), SR1 (500 nmol/L, Alichem) and UM729 (500 nmol/L, IRIC).

Cell culture

Cell lines were purchased from the ATCC or donated from collaborators. OCI-AML5 cell line was cultured in αMEM, 10% heat-inactivated FBS supplemented with 10 ng/mL GM-CSF, K562 cell line was maintained in IMDM 10% heat-inactivated FBS, HEK293 and HEK293T were kept in DMEM, 10% heat-inactivated FBS. Cells were maintained at 37°C in 5% CO_2 atmosphere. K562 cell lines WT or heterozygous for *SF3B1* (K700E/+) were purchased from Horizon Discovery (#HD 181-012 and #HD PAR-131) and maintained in IMDM supplemented with 10% heat-inactivated FBS. For experiments performed under hypoxic conditions, cells were incubated in regular media and incubated at 1% O_2 for 4 days.

Dose-Response assays in cell lines

Chemical screens were performed as in primary AML samples with a few modifications: 300 cells per well were seeded in 50 μL of media, compounds were added in serial dilution (8 dilutions, 1:4, 10 μM down to 0.6 nM) and cell viability was evaluated after 4 days in culture.

Metal binding assays

Metal chelation property of compounds was monitored by UV-visible spectroscopy analysis using a Biochrom WPA Lightwave II+ spectrophotometer ranging from 200 to 600 nm. Molecules were solubilized in ethanol at a concentration of 40 μM and 1 equivalent of various metal ions ($CuCl_2$, $FeCl_2$, $ZnCl_2$) in ethanol were added. All absorbance spectra were recorded 30 minutes after addition in a quartz cell of 1 cm optical path length at room temperature.

Immunoblot analysis

For γH2AX and p53 immunoblots, total proteins were extracted using Laemmli buffer (125 mM

Tris-HCl pH7.4, 4% SDS, 20% Glycerol, 10% 2-mercaptoethanol, 0.004% bromophenol blue). Proteins were resolved by SDS-PAGE, transferred onto PVDF membrane, blocked with 5% milk and probed with primary (overnight, 4C) and secondary (1h, room temperature) antibodies. For FDX1, ABCB7, POLD1, LIAS, DLAT and lipoic acid immunoblots, the same protocol was applied with the exception that total proteins were extracted using RIPA buffer (20 mM Tris-HCl pH7.4, 150 mM NaCl, 5 mM MgCl2, 5 mM EGTA, 60 mM β-glycerophosphate, 0.1% NP40, 0.1% Triton X-114, 1 mM DTT) supplemented with protease inhibitors (cOmplete, EDTA-free protease inhibitor tablets), and quantified by the bicinchoninic acid (BCA) method using a BSA standard curve. Primary antibodies used are as follow: γH2A.X (Millipore 05-636), p53 phospho-ser15 (Cell Signaling Technology 9284S), FDX1 (Novus Biologicals NBP1-89227), ABCB7 (Novus Biologicals NBP2-21600), POLD1 (Abcam ab186407), LIAS (ProteinTech 11577-1-AP), DLAT (Cell Signaling Technology 12362), lipoic acid (Abcam ab58724) and alpha-tubulin (Cell Signaling Technology 2144S). Secondary antibodies used are as follow: anti-mouse HRP (Jackson ImmunoResearch 115-035-146) and anti-rabbit HRP (Jackson ImmunoResearch 111-035-144).

Immunofluorescence

OCI-AML5 cells were treated 16 hours with compounds, washed in PBS and plated onto poly-l-lysine coated Ibidi microscopy chambers. Cells were fixed with 1% paraformaldehyde for 8 min at room temperature and were then washed in PBS. After blocking in PBS with 1% bovine serum albumin and 0.25% Triton, cells were stained for 2 h at room temperature with DLAT antibody (Cell Signaling Technology 12362, dilution 1:100). Cells were further washed and stained with Alexa Fluor 647 anti-mouse secondary antibody (ThermoFisher A-21236) and finally counterstained with DAPI. Following immunostaining, images were captured on a Zeiss LSM 880 confocal microscope driven by ZEN software at 60X. Total DLAT signal was quantified using Image J software (NIH) and is reported as total DLAT intensity divided by the number of cells in each image.

Synergy

Dose response of copper (33 to 33500 nM) or Pladienolide B (0.00005 to 0.05 nM) were assessed in combination with increasing concentrations of UM4118 (5 to 5000 nM or 0.5 to 500 nM, respectively) in 384-well plates to determine synergistic interactions. 300 OCI-AML5 cells per well were seeded in 50 μL of media, and cell viability was evaluated after 4 days in culture by CellTiterGlo assay (Promega). Synergy scores were calculated according to the Bliss method.

Knockdown experiments by shRNA

Lentiviral vectors carrying shRNAs targeting *ABCB7, GLRX3, NFS1, MMS19, NFU1, DLAT* or *CTR1* gene were generated by cloning shRNA sequences into MNDU vectors comprising miR-E sequences (*62*). Control vector (shRNA ctrl) contained shRNA targeting Renilla luciferase. Sequences of the 22-mer guide shRNAs are as follow: *ABCB7*: TTGTAATAAATAGTATTATGGA; *GLRX3*: TTCATAAACAGCATGCAGGGGG; *NFS1*: AATTATTAGAATAACTGGTGGA; *MMS19*: TCATAGAACAGTATCAGGTGTA; *NFU1*: TATATATTATCAACAAGTCTGA; *DLAT*: TTCATTTACAATGAACTACCAG; *CTR1 #1*: TAATGTTAAGTTTGAGTTCTGG; *CTR1 #2*: TTGGTATAACGTATCACATCTA and *CTR1 #3*: TAATCAAAGTGTTAAGTGGGAA. HEK293T cells were transfected with 5 μg lentiviral plasmid, 3.3 μg PAX2 packaging plasmid and 1 μg VSV-G envelope plasmid using 20 μL of JetPrime Transfection reagent (PolyPlus Transfection), according to manufacturer's directions to

produce lentiviruses. Viral supernatants were collected after 48 hours, filtered and used to infect cell lines at a multiplicity of infection of 5 in media supplemented with 10 ng/mL polybrene for 48 hours. Infection efficiency, as determined by the percentage of GFP positive cells, was monitored by flow cytometry using a BD FACSCanto II and infected cells were selected with puromycin.

Oxygen consumption rate analysis

Oxygen consumption rate (OCR) in OCI-AML5 cells was measured using a 96-well Seahorse Bioanalyzer XFe96 according to the manufacturer's instructions (Agilent Technologies). OCI-AML5 cells were seeded into Seahorse 96-well plates pre-coated for 3 hours with polylysine (Sigma-Aldrich, P4707) at a density of 100000 cells/well in Seahorse XF Base Medium (Agilent Technologies #103575-100). Cells were treated for 24 hours with compounds or DMSO prior to analysis (n=6 for each conditions). For OCR measurements, compounds were acutely injected in media at a final concentration of 1 μM for Oligomycin, 0.5 μM for FCCP and 0.5 μM for Rotenone/Antimycin A.

Total proteome

10 million OCI-AML5 cells (seeding density of 0.5 M / mL) were treated in triplicate with DMSO or 300 nM UM4118 for 16 hours in media supplemented with 1 μM copper. Collected cells were washed in PBS and cell pellets lysed in Triton X-100 buffer (10 mM PIPES pH 7.4, 0.5% Triton X-100, 300 mM Sucrose, 100 mM NaCl, 3 mM MgCl2, 0.5 mM EDTA, protease inhibitors). A total of 100 μg of proteins for each sample was precipitated and washed using MeOH/CHCl3. Samples were reconstituted in 50 mM ammonium bicarbonate with 10 mM TCEP (Tris(2-carboxyethyl) phosphine hydrochloride; Thermo Fisher Scientific), and vortexed for 1 h at 37°C. Chloroacetamide (Sigma-Aldrich) was added for alkylation to a final concentration of 55 mM. Samples were vortexed for another hour at 37°C. One microgram of trypsin was added, and digestion was performed for 8 h at 37°C. Samples were dried down and solubilized in 5% ACN-4% formic acid (FA). The samples were loaded on a 1.5 μl pre-column (Optimize Technologies, Oregon City, OR). For each run, 1 μg of peptides was separated on an home-made reversed-phase column (150-μm i.d. by 200 mm) with a 116-min gradient from 10 to 30% ACN-0.2% FA and a 600-nl/min flow rate on an Easy nLC-1200 connected to a Exploris 480 (Thermo Fisher Scientific, San Jose, CA) with the FAIMS interface. Each proteome was analyzed with 4 separate LC-MS/MS runs acquired with a different set of compensation voltages (CV) and a different m/z range: run1 was performed with the CV set-44V-54V-64V-74V from m/z 350 to 453, run 2 was performed with the CV set -40V-48V-56V-64V-72V-80V from m/z 451 to 542, run 3 was performed with the CV set -40V-48V-56V-64V-72V-80V from m/z 540 to 661, run 4 was performed with the CV set -35V-46V-57V from m/z 659 to 890. Each full MS spectrum acquired at a resolution of 120,000 was followed by tandem-MS (MS-MS) spectra acquisition on the most abundant multiply charged precursor ions for 3s. Tandem-MS experiments were performed using higher energy collision dissociation (HCD) at a collision energy of 34% with an AGC of 50% a resolution of 45,000 and an injection time of 100 ms. The data were processed using PEAKS X Pro (Bioinformatics Solutions, Waterloo, ON) and a Uniprot human database (20366 entries) with trypsin as the enzyme. Mass tolerances on precursor and fragment ions were 10 ppm and 0.01 Da, respectively. Fixed modification was carbamidomethyl (C). Variable selected posttranslational modifications were acetylation (N-ter), oxidation (M), deamidation (NQ), phosphorylation (STY).

Differential Enrichment analysis of Proteomics data (DEP) package (*63*) in R was used to analyze

the data (excluding proteins identified based on a single peptide), including data filtering, normalization, imputation of missing values and statistical testing of differentially expressed proteins. Analysis for gene set enrichment was done using STRING (*64*).

Cell proliferation assay

100.000 OCI-AML5 cells, previously infected with lentivirus expressing the indicated shRNAs, were seeded in 48-well plates at day 0 and treated with DMSO or 150 nM UM4118. Every 2 or 3 days, cells were counted by flow cytometry (BD FACSCanto II) using CountBright absolute counting beads. Cells were kept at a density of 0.2 M / mL through the experiment. Counts were normalized to DMSO treated cells to take in consideration any proliferative impact of each individual shRNAs.

Clonogeny assay

K562 cell lines WT or carrying the *SF3B1* K700E heterozygous mutation were treated 48 hours with DMSO or 20 nM of the copper ionophores (elescomol or UM4118). 1500 cells were then seeded in triplicate in IMDM 1% methylcellulose supplemented by 20% heat-inactivated FBS, 1% deionized BSA, 2 mM Glutamine, 200 µg/mL Holo-transferrin, 10^{-4} M b-mercaptoethanol, 100 ng/mL SCF, 10 ng/mL IL-3, 10 ng/mL GM-CSF, 3 U/mL Epo, 10 ng/mL IL-6 and 50 ng/mL Tpo. Colonies were counted 10 days after seeding.

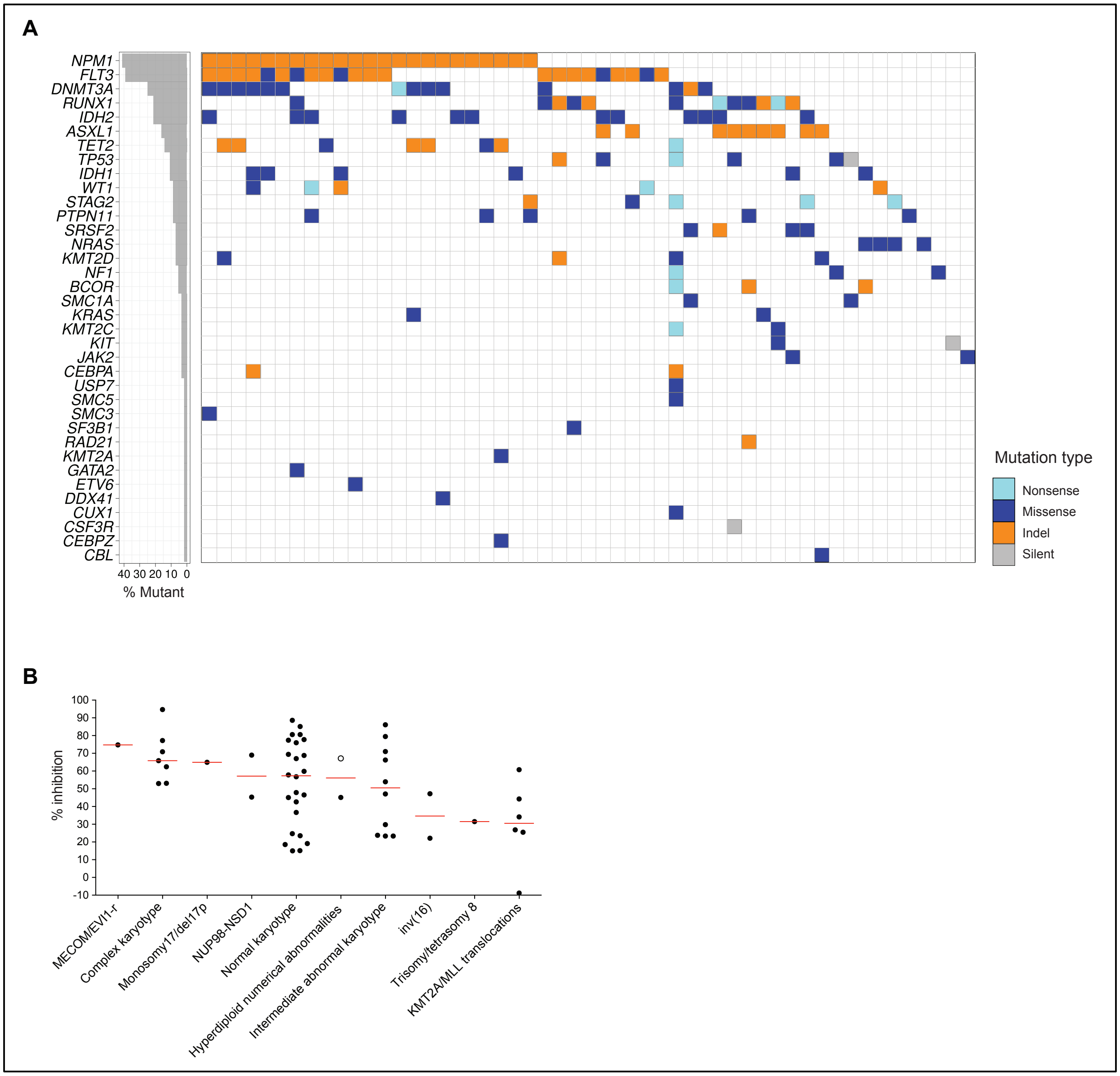

<u>Figure S1:</u> Primary AML cohort and results of the discovery screen.

(**A**) Mutation heatmap of the 56 primary AML patient samples used in the discovery screen (3 specimens without mutations are not represented). Genes (y-axis) are ordered based on their mutation frequencies while AML specimens are represented in the x-axis. (**B**) Dot plot distribution of S767 associated percentage of inhibition across the different AML specimen subgroups (discovery screen). Normalized to DMSO control treatment, empty dot represents *SF3B1*-mutated sample, median is represented in red.

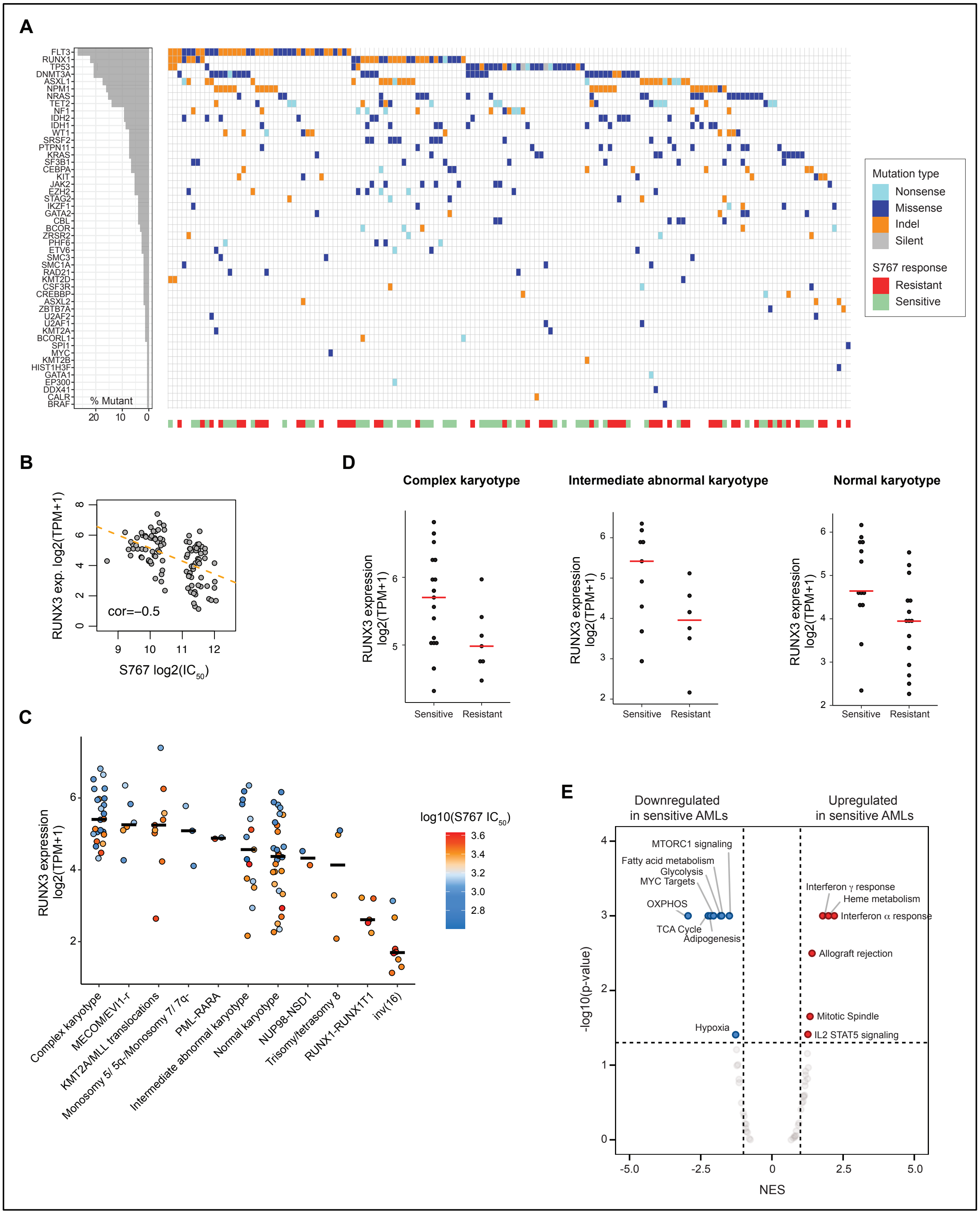

<u>Figure S2:</u> Primary AML cohort and results of the validation screen.

(**A**) Mutation heatmap of the primary AML patient samples used in the validation screen (specimens without mutations are not represented). Genes (y-axis) are ordered based on their

mutation frequencies while AML specimens are represented in the x-axis. Sensitive versus resistant AMLs were splited according to tier1 and tier3 of their IC_{50} values. (**B**) Anti-correlation observed between *RUNX3* mRNA expression and S767 IC_{50} values obtained in primary specimens. (**C**) Dot plot representation of *RUNX3* mRNA expression in primary AMLs used in the validation screen. S767 sensitivity is color-coded and median is depicted in black. (**D**) Dot plot representation of *RUNX3* mRNA expression in complex karyotype, intermediate abnormal karyotype and normal karyotype AMLs tested in the validation screen according to their sensitivity to S767. (**E**) Gene set enrichment analysis (GSEA) comparing transcriptomic signatures of tier1 (sensitive) versus tier3 (resistant) primary AMLs included in the validation screen.

A

		Analog series	IC_{50} in AML5	Ratio IC_{50} shRNA ctrl / ABCB7	γH2AX
S767	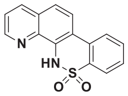	sulfonamide	1067 nM	2.2	positive
UM4005	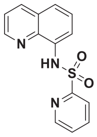	sulfonamide	248 nM	1.2	positive
UM3871	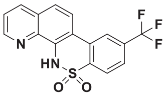	sulfonamide	646 nM	2.3	positive
UM4207	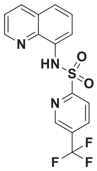	sulfonamide	226 nM	1	positive
UM4186	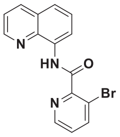	picolinamide	212 nM	3.5	negative
UM4178	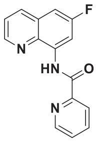	picolinamide	143 nM	3.2	negative
UM4199	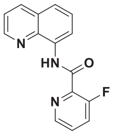	picolinamide	57 nM	2.8	negative
UM4118	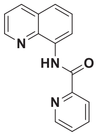	picolinamide	40 nM	3.2	negative

B

UM4118

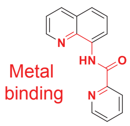

IC_{50} in AML5: 49 nM

UM4181

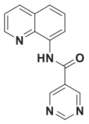

IC_{50} in AML5: > 10000 nM

UM4185

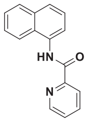

IC_{50} in AML5: > 10000 nM

UM4206

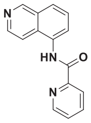

IC_{50} in AML5: > 10000 nM

Figure S3: Structure-activity relationship (SAR) related to S767 optimization.
(**A**) Representative examples of the S767 analogs including the sulfonamide and picolinamide series. IC_{50} values in OCI-AML5 are depicted, as well as the ratio of IC_{50} values obtained in OCI-

AML5 stably expressing an shRNA control versus an shRNA targeting *ABCB7*. Molecules with a ratio > 2 were considered on-target. (**B**) Structures of UM4118 and example of analogs carrying structural modifications that abrogate metal binding properties of the molecules. Elements that confer metal binding are depicted in red in UM4118 structure. IC_{50} values were determined in OCI-AML5 cells (highest dose tested = 10 μM).

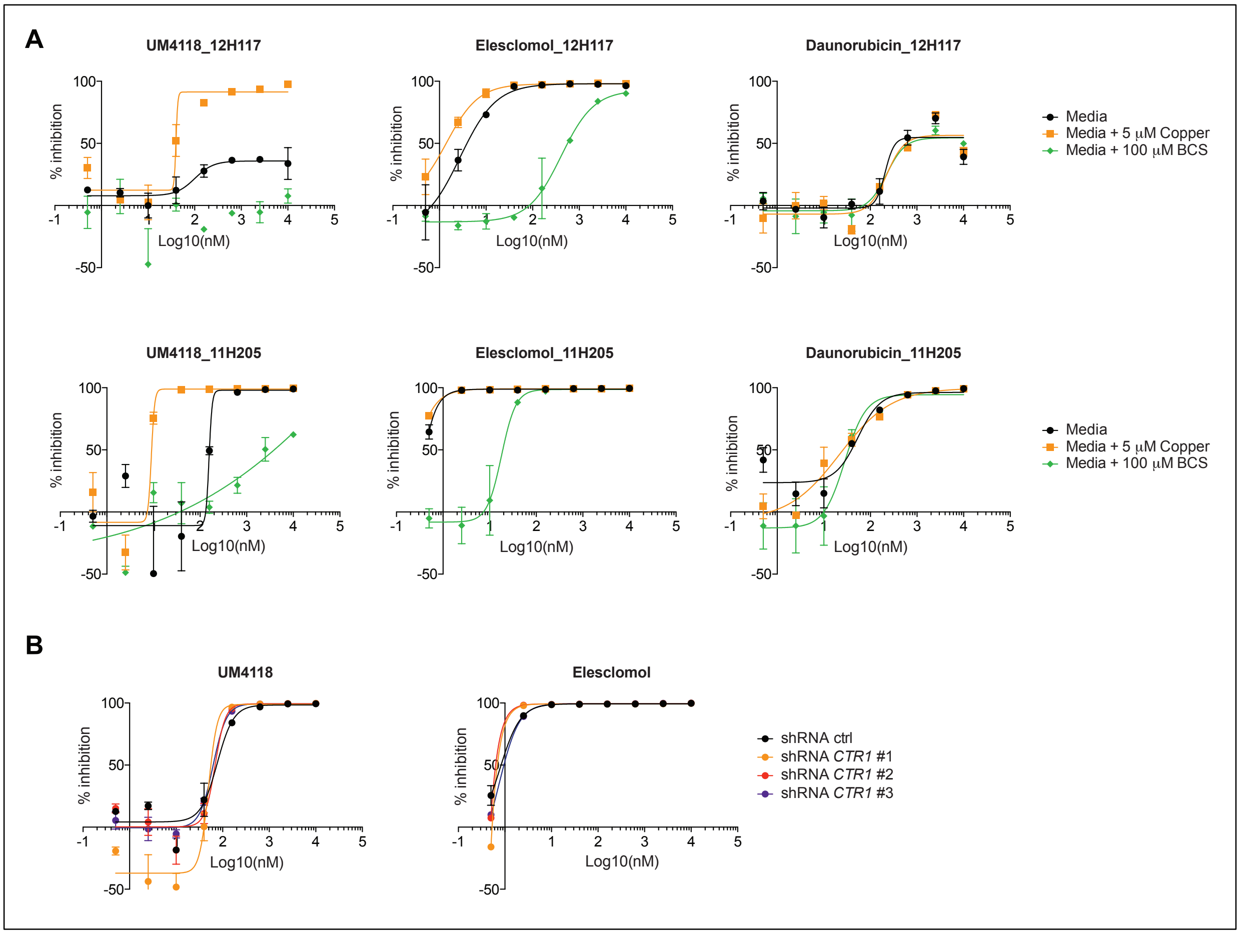

<u>Figure S4:</u> Copper-dependent activity of UM4118 molecule.

(**A**) Dose-response curves obtained in primary AML samples 12H117 (complex karyotype) and 11H205 (*MECOM/EVI1*-rearranged) exposed to indicated compounds in regular AML media, supplemented with 5 µM copper or 100 µM BCS (error bars indicate SD of technical duplicates). Daunorubicin is used as a negative control. (**B**) Representative dose-response curves for UM4118 and elesclomol obtained in OCI-AML5 cells constitutively expressing shRNAs targeting *CTR1* (error bars indicate SD of technical duplicates).

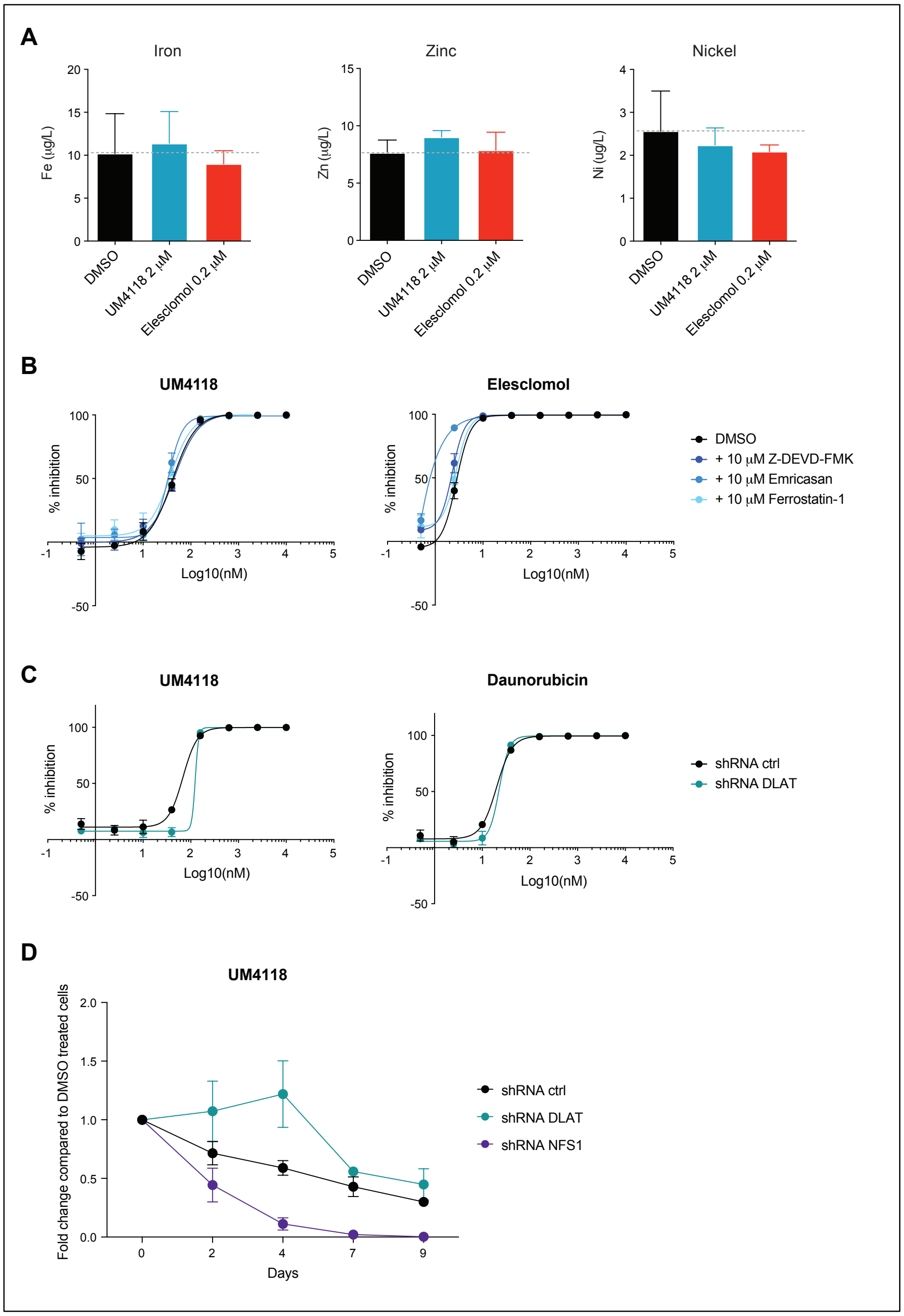

<u>Figure S5:</u> UM4118 induces cell death by cuproptosis.

(**A**) Intra-mitochondrial metal quantification (ICP-MS) of HEK293 cells exposed 3 hours to indicated compounds. Data is represented as mean +/- SD (n=3, unpaired t test compared to DMSO condition). (**B**) Representative dose-response curves for UM4118 and elesclomol obtained in OCI-AML5 cells pre-treated with 10 µM Ferrostatin-1 (ferroptosis inhibitor), Z-DEVD-FMK (caspase 3 inhibitor) or Emricasan (pan caspase inhibitor). Error bars indicate SD of technical duplicates. (**C**) Representative dose-response curves for UM4118 obtained in OCI-AML1 cells constitutively expressing an shRNA targeting *DLAT* (error bars indicate SD of technical duplicates). Daunorubicin is used as a negative control. (**D**) Proliferation assay of OCI-AML5 cells

constitutively expressing shRNAs targeting *DLAT* or *NFS1* in the presence of 150 nM UM4118 (n=2, mean +/- SD). Cellular counts were normalized to DMSO treated cells.

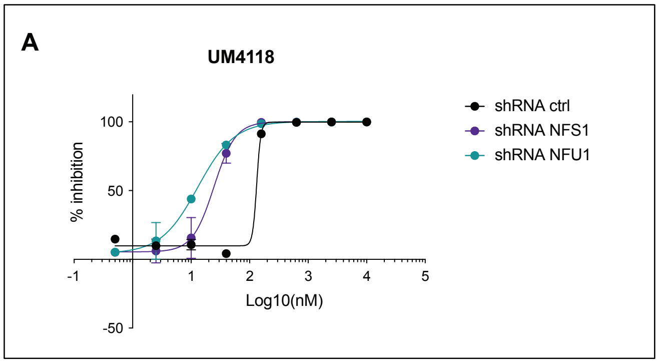

<u>Figure S6:</u> ISC deficiency potentiates UM4118 cytotoxicity.

(**A**) Representative dose-response curves for UM4118 obtained in OCI-AML5 cells constitutively expressing shRNAs targeting *NFS1* or *NFU1* (error bars indicate SD of technical duplicates).

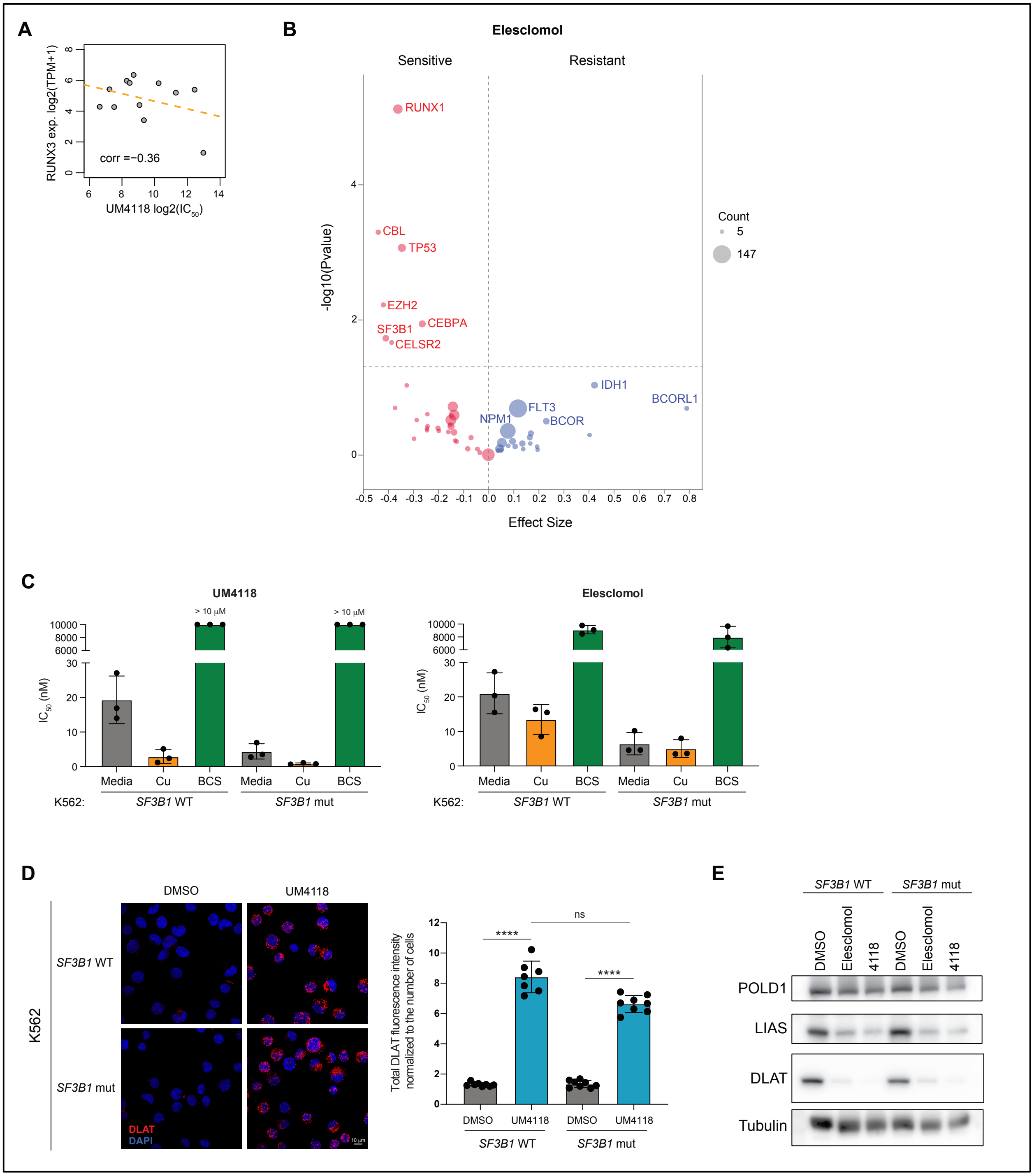

Figure S7: ***SF3B1*** **mutated cells are sensitivite to copper ionophores.**
(**A**) Anti-correlation observed between *RUNX3* mRNA expression and UM4118 IC_{50} values in primary AML specimens. (**B**) Volcano plot representation of the statistical associations between elesclomol sensitivity and the mutational status of primary AML specimens of the BEAT AML cohort (data extracted from Vizome, default settings). (**C**) IC_{50} values of UM4118 and elesclomol

determined in regular media, in media supplemented with 2 μM copper (Cu) or in the presence of the copper chelator BCS (100 μM) in K562 *SF3B1* WT or mutated cells. Data is represented as mean +/- SD (n=3). (**D**) Representative images (left) of DLAT staining (red) in K562 *SF3B1* WT or mutated cells exposed 16 hours to 300 nM UM4118 in media supplemented with 1 μM copper. Nuclei are counterstained with DAPI (blue), scale bar = 10 μm. Quantification (right) of total DLAT signal divided per the number of cells in each image is depicted (unpaired t test). (**E**) Immunoblot analysis of POLD1, LIAS and DLAT proteins on K562 *SF3B1* WT or mutated cells cultured in media supplemented with 1 μM copper and exposed 16 hours to 30 nM elesclomol, 300 nM UM4118 or DMSO. Tubulin is used as a loading control.

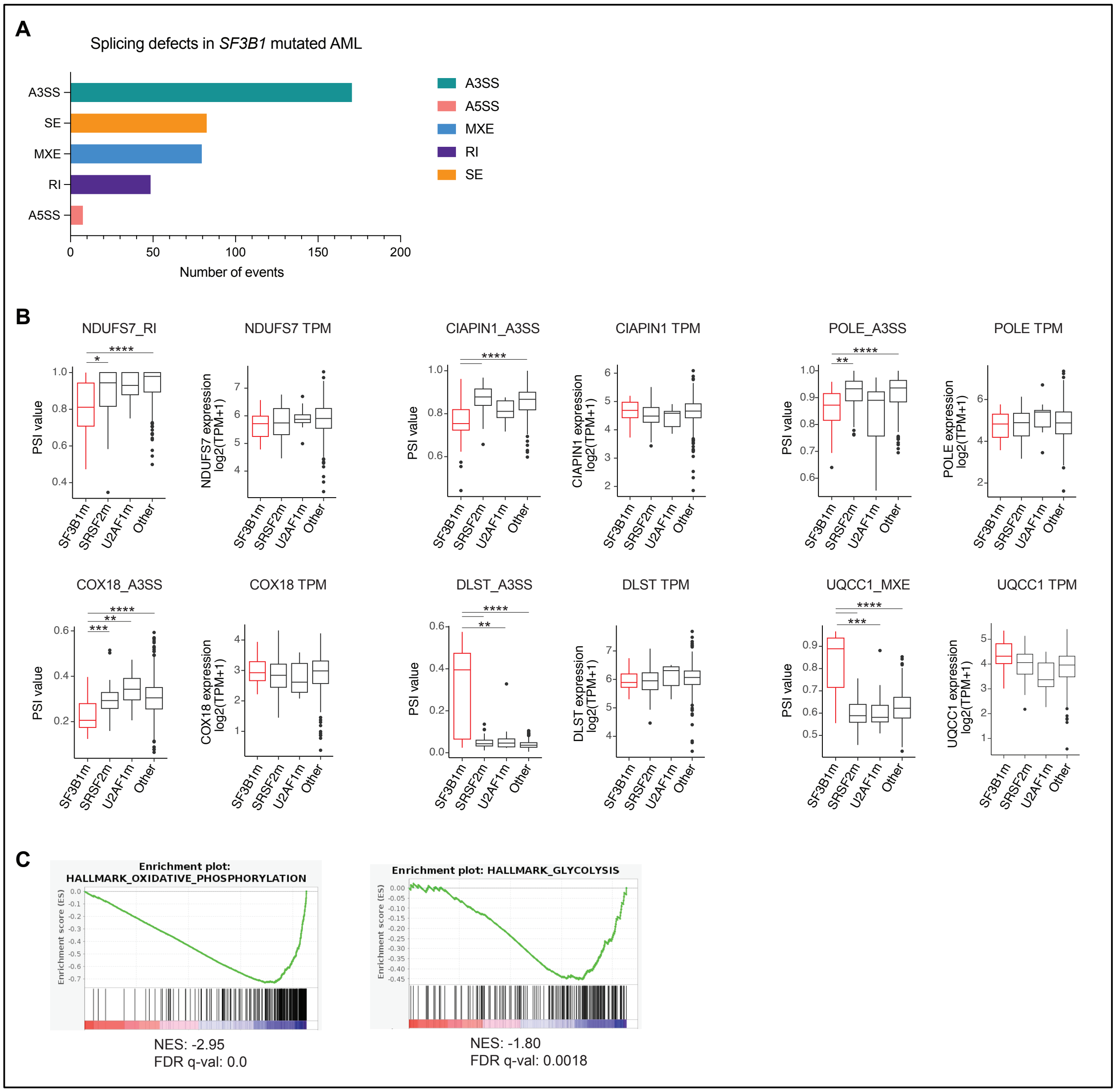

<u>Figure S8:</u> Splicing defects in *SF3B1*-mutated primary AMLs.
(**A**) Distribution of the splicing alterations found in *SF3B1*-mutated primary AMLs compared to WT. Only events presenting a significant difference of PSI (ΔPSI ≥ 5%, p < 0.01 Mann-Whitney U test) between *SF3B1*-mutated samples (n=20) and control AMLs (n=671) were kept. (**B**) Box plot representation of the percent-splice-in (PSI) value and total mRNA expression in TPM (transcripts per million) of representative genes in primary AML specimens with or without mutations in the splicing factors *SF3B1*, *SRSF2* (n mutated = 62) or *U2AF1* (n mutated = 11) (Mann-Whitney U test). (**C**) Gene set enrichment analysis (GSEA) plot comparing transcriptomic signatures of tier1 (sensitive) versus tier3 (resistant) primary AMLs included in the validation screen. A3SS: alternative 3’ splice site, A5SS: alternative 5’ splice site, SE: skipped exon, MXE: mutually exclusive exon, RI: retained intron.

Other Supplementary Materials for this manuscript include the following:

Tables S1 to S8 in excel documents

Table S1: Data for S767 molecule in discovery and validation screens (primary AML specimens).
Table S2: Differential expression analysis between most sensitive (tier 1) and most resistant (tier 3) primary AML specimens in response to S767.
Table S3: Transcriptome data of OCI-AML5 cells exposed 24 hours to 1 μM of S767.
Table S4: Whole genome CRISPR/Cas9 loss-of-function screen results obtained in cells exposed to S767.
Table S5: Aberrant splicing events identified in *SF3B1*-mutated primary AML patient samples compared to WT.
Table S6: *ABCB7* mRNA expression and PSI/Ψ (Percent Spliced-In) value determined in primary AML patient samples.
Table S7: Whole genome CRISPR/Cas9 loss-of-function screen results obtained in cells exposed to UM4118.
Table S8: Total proteome data of OCI-AML5 cells exposed 16 hours to 300 nM of UM4118.

REFERENCES AND NOTES

1. B.-E. Kim, T. Nevitt, D. J. Thiele, Mechanisms for copper acquisition, distribution and regulation. *Nat. Chem. Biol.* **4**, 176–185 (2008).

2. L. M. Ruiz, A. Libedinsky, A. A. Elorza, Role of copper on mitochondrial function and metabolism. *Front. Mol. Biosci.* **8**, 711227 (2021).

3. V. C. Shanbhag, N. Gudekar, K. Jasmer, C. Papageorgiou, K. Singh, M. J. Petris, Copper metabolism as a unique vulnerability in cancer. *Biochim Biophys Acta Mol Cell Res.* **1868**, 118893 (2021).

4. S. K. Gupta, V. K. Shukla, M. P. Vaidya, S. K. Roy, S. Gupta, Serum and tissue trace elements in colorectal cancer. *J. Surg. Oncol.* **52**, 172–175 (1993).

5. S. Ishida, P. Andreux, C. Poitry-Yamate, J. Auwerx, D. Hanahan, Bioavailable copper modulates oxidative phosphorylation and growth of tumors. *Proc. Natl. Acad. Sci. U.S.A.* **110**, 19507–19512 (2013).

6. K. Sharma, D. K. Mittal, R. C. Kesarwani, V. P. Kamboj, Diagnostic and prognostic significance of serum and tissue trace elements in breast malignancy. *Indian J. Med. Sci.* **48**, 227–232 (1994).

7. A. Steinbrueck, A. C. Sedgwick, J. T. Brewster, K. C. Yan, Y. Shang, D. M. Knoll, G. I. Vargas-Zúñiga, X. P. He, H. Tian, J. L. Sessler, Transition metal chelators, pro-chelators, and ionophores as small molecule cancer chemotherapeutic agents. *Chem. Soc. Rev.* **49**, 3726–3747 (2020).

8. P. Tsvetkov, S. Coy, B. Petrova, M. Dreishpoon, A. Verma, M. Abdusamad, J. Rossen, L. Joesch-Cohen, R. Humeidi, R. D. Spangler, J. K. Eaton, E. Frenkel, M. Kocak, S. M. Corsello, S. Lutsenko, N. Kanarek, S. Santagata, T. R. Golub, Copper induces cell death by targeting lipoylated TCA cycle proteins. *Science* **375**, 1254–1261 (2022).

9. S. Ciofi-Baffoni, V. Nasta, L. Banci, Protein networks in the maturation of human iron-sulfur proteins. *Metallomics* **10**, 49–72 (2018).

10. R. Lill, From the discovery to molecular understanding of cellular iron-sulfur protein biogenesis. *Biol. Chem.* **401**, 855–876 (2020).

11. R. Shi, W. Hou, Z.-Q. Wang, X. Xu, Biogenesis of iron-sulfur clusters and their role in dna metabolism. *Front. Cell Dev. Biol.* **9**, 735678 (2021).

12. P. Csere, R. Lill, G. Kispal, Identification of a human mitochondrial ABC transporter, the functional orthologue of yeast Atm1p. *FEBS Lett.* **441**, 266–270 (1998).

13. R. Allikmets, W. H. Raskind, A. Hutchinson, N. D. Schueck, M. Dean, D. M. Koeller, Mutation of a putative mitochondrial iron transporter gene (ABC7) in X-linked sideroblastic anemia and ataxia (XLSA/A). *Hum. Mol. Genet.* **8**, 743–749 (1999).

14. S. Bekri, G. Kispal, H. Lange, E. Fitzsimons, J. Tolmie, R. Lill, D. F. Bishop, Human ABC7 transporter: Gene structure and mutation causing X-linked sideroblastic anemia with ataxia with disruption of cytosolic iron-sulfur protein maturation. *Blood* **96**, 3256–3264 (2000).

15. C. Pondarré, B. B. Antiochos, D. R. Campagna, S. L. Clarke, E. L. Greer, K. M. Deck, A. McDonald, A. P. Han, A. Medlock, J. L. Kutok, S. A. Anderson, R. S. Eisenstein, M. D. Fleming, The mitochondrial ATP-binding cassette transporter Abcb7 is essential in mice and participates in cytosolic iron-sulfur cluster biogenesis. *Hum. Mol. Genet.* **15**, 953–964 (2006).

16. S. Alsafadi, A. Houy, A. Battistella, T. Popova, M. Wassef, E. Henry, F. Tirode, A. Constantinou, S. Piperno-Neumann, S. Roman-Roman, M. Dutertre, M. H. Stern, Cancer-associated SF3B1 mutations affect alternative splicing by promoting alternative branchpoint usage. *Nat. Commun.* **7**, 10615–12 (2016).

17. A. K. Kesarwani, O. Ramirez, A. K. Gupta, X. Yang, T. Murthy, A. C. Minella, M. M. Pillai, Cancer-associated SF3B1 mutants recognize otherwise inaccessible cryptic 3′ splice sites within RNA secondary structures. *Oncogene* **36**, 1123–1133 (2017).

18. E. Papaemmanuil, M. Cazzola, J. Boultwood, L. Malcovati, P. Vyas, D. Bowen, A. Pellagatti, J. S. Wainscoat, E. Hellstrom-Lindberg, C. Gambacorti-Passerini, A. L. Godfrey, I. Rapado, A. Cvejic, R. Rance, C. McGee, P. Ellis, L. J. Mudie, P. J. Stephens, S. McLaren, C. E. Massie, P. S. Tarpey, I. Varela, S. Nik-Zainal, H. R. Davies, A. Shlien, D. Jones, K. Raine, J. Hinton, A. P. Butler, J. W. Teague, E. J. Baxter, J. Score, A. Galli, M. G. Della Porta, E. Travaglino, M. Groves, S. Tauro, N. C. Munshi, K. C. Anderson, A. el-Naggar, A. Fischer, V. Mustonen, A. J. Warren, N. C. Cross, A. R. Green, P. A. Futreal, M. R. Stratton, P. J. Campbell, Chronic Myeloid Disorders Working Group of the International Cancer Genome Consortium, Somatic SF3B1 mutation in myelodysplasia with ring sideroblasts. *N. Engl. J. Med.* **365**, 1384–1395 (2011).

19. H. Dolatshad, A. Pellagatti, F. G. Liberante, M. Llorian, E. Repapi, V. Steeples, S. Roy, L. Scifo, R. N. Armstrong, J. Shaw, B. H. Yip, S. Killick, R. Kušec, S. Taylor, K. I. Mills, K. I. Savage, C. W. J. Smith, J. Boultwood, Cryptic splicing events in the iron transporter ABCB7 and other key target genes in SF3B1-mutant myelodysplastic syndromes. *Leukemia* **30**, 2322–2331 (2016).

20. M. Nikpour, C. Scharenberg, A. Liu, S. Conte, M. Karimi, T. Mortera-Blanco, V. Giai, M. Fernandez-Mercado, E. Papaemmanuil, K. Högstrand, M. Jansson, I. Vedin, J. Stephen Wainscoat, P. Campbell, M. Cazzola, J. Boultwood, A. Grandien, E. Hellström-Lindberg, The transporter ABCB7 is a mediator of the phenotype of acquired refractory anemia with ring sideroblasts. *Leukemia* **27**, 889–896 (2013).

21. Y. Shiozawa, L. Malcovati, A. Gallì, A. Sato-Otsubo, K. Kataoka, Y. Sato, Y. Watatani, H. Suzuki, T. Yoshizato, K. Yoshida, M. Sanada, H. Makishima, Y. Shiraishi, K. Chiba, E. Hellström-Lindberg, S. Miyano, S. Ogawa, M. Cazzola, Aberrant splicing and defective mRNA production induced by somatic spliceosome mutations in myelodysplasia. *Nat. Commun.* **9**, 3649–16 (2018).

22. C. A. Clough, J. Pangallo, M. Sarchi, J. O. Ilagan, K. North, R. Bergantinos, M. C. Stolla, J. Naru, P. Nugent, E. Kim, D. L. Stirewalt, A. R. Subramaniam, O. Abdel-Wahab, J. L. Abkowitz, R. K. Bradley, S. Doulatov, Coordinated missplicing of TMEM14C and ABCB7 causes ring

sideroblast formation in SF3B1-mutant myelodysplastic syndrome. *Blood* **139**, 2038–2049 (2022).

23. A. C. Menezes, R. Jones, A. Shrestha, R. Nicholson, A. Leckenby, A. Azevedo, S. Davies, S. Baker, A. F. Gilkes, R. L. Darley, A. Tonks, Increased expression of RUNX3 inhibits normal human myeloid development. *Leukemia* **36**, 1769–1780 (2022).

24. T. Bertomeu , J. Coulombe-Huntington , A. Chatr-Aryamontri , K. G. Bourdages, E. Coyaud, B. Raught, Y. Xia, M. Tyers, A high-resolution genome-wide CRISPR/Cas9 viability screen reveals structural features and contextual diversity of the human cell-essential proteome. *Mol. Cell. Biol.* **38**, 387–24 (2017).

25. M. Nagai, N. H. Vo, L. Shin Ogawa, D. Chimmanamada, T. Inoue, J. Chu, B. C. Beaudette-Zlatanova, R. Lu, R. K. Blackman, J. Barsoum, K. Koya, Y. Wada, The oncology drug elesclomol selectively transports copper to the mitochondria to induce oxidative stress in cancer cells. *Free Radic. Biol. Med.* **52**, 2142–2150 (2012).

26. R. K. Blackman, K. Cheung-Ong, M. Gebbia, D. A. Proia, S. He, J. Kepros, A. Jonneaux, P. Marchetti, J. Kluza, P. E. Rao, Y. Wada, G. Giaever, C. Nislow, Mitochondrial electron transport is the cellular target of the oncology drug elesclomol. *PLOS One* **7**, e29798 (2012).

27. C. Molinaro, A. Martoriati, L. Pelinski, K. Cailliau, Copper Complexes as Anticancer Agents Targeting Topoisomerases I and II. *Cancers* **12**, 2863 (2020).

28. R. Galindo-Murillo, J. C. García-Ramos, L. Ruiz-Azuara, T. E. Cheatham, F. Cortés-Guzmán, Intercalation processes of copper complexes in DNA. *Nucleic Acids Res.* **43**, 5364–5376 (2015).

29. N. Z. Fantoni, T. Brown, A. Kellett, DNA-targeted metallodrugs: An untapped source of artificial gene editing technology. *Chembiochem* **22**, 2184–2205 (2021).

30. P. R. Joshi, S. Sadre, X. A. Guo, J. G. McCoy, V. K. Mootha, Lipoylation is dependent on the ferredoxin FDX1 and dispensable under hypoxia in human cells. *J. Biol. Chem.*, **299**, 105075 (2023),.

31. M. B. Dreishpoon N. R. Bick, B. Petrova, D. M. Warui, A. Cameron, S. J. Booker, N. Kanarek, T. R. Golub, P. Tsvetkov, FDX1 regulates cellular protein lipoylation through direct binding to LIAS. *J. Biol. Chem.*, 105046 (2023).

32. J. W. Tyner, C. E. Tognon, D. Bottomly, B. Wilmot, S. E. Kurtz, S. L. Savage, N. Long, A. R. Schultz, E. Traer, M. Abel, A. Agarwal, A. Blucher, U. Borate, J. Bryant, R. Burke, A. Carlos, R. Carpenter, J. Carroll, B. H. Chang, C. Coblentz, A. d'Almeida, R. Cook, A. Danilov, K. H. T. Dao, M. Degnin, D. Devine, J. Dibb, D. K. Edwards V, C. A. Eide, I. English, J. Glover, R. Henson, H. Ho, A. Jemal, K. Johnson, R. Johnson, B. Junio, A. Kaempf, J. Leonard, C. Lin, S. Q. Liu, P. Lo, M. M. Loriaux, S. Luty, T. Macey, J. MacManiman, J. Martinez, M. Mori, D. Nelson, C. Nichols, J. Peters, J. Ramsdill, A. Rofelty, R. Schuff, R. Searles, E. Segerdell, R. L. Smith, S. E. Spurgeon, T. Sweeney, A. Thapa, C. Visser, J. Wagner, K. Watanabe-Smith, K. Werth, J. Wolf, L. White, A. Yates, H. Zhang, C. R. Cogle, R. H. Collins, D. C. Connolly, M. W. Deininger, L. Drusbosky, C. S. Hourigan, C. T. Jordan, P. Kropf, T. L. Lin, M. E. Martinez, B. C. Medeiros, R. R. Pallapati, D. A. Pollyea, R. T. Swords, J. M. Watts, S. J. Weir, D. L. Wiest, R. M. Winters, S. K. McWeeney, B. J. Druker, Functional genomic landscape of acute myeloid leukaemia. *Nature* **562**, 526–531 (2018).

33. L. Macomber, J. A. Imlay, The iron-sulfur clusters of dehydratases are primary intracellular targets of copper toxicity. *Proc. Natl. Acad. Sci. U.S.A.* **106**, 8344–8349 (2009).

34. D. Brancaccio, A. Gallo, M. Piccioli, E. Novellino, S. Ciofi-Baffoni, L. Banci, [4Fe-4S] Cluster assembly in mitochondria and its impairment by copper. *J. Am. Chem. Soc.* **139**, 719–730 (2017).

35. S. Chillappagari, A. Seubert, H. Trip, O. P. Kuipers, M. A. Marahiel, M. Miethke, Copper stress affects iron homeostasis by destabilizing iron-sulfur cluster formation in Bacillus subtilis. *J. Bacteriol.* **192**, 2512–2524 (2010).

36. W. B. Dalton, E. Helmenstine, N. Walsh, L. P. Gondek, D. S. Kelkar, A. Read, R. Natrajan, E. S. Christenson, B. Roman, S. das, L. Zhao, R. D. Leone, D. Shinn, T. Groginski, A. K. Madugundu, A. Patil, D. J. Zabransky, A. Medford, J. Lee, A. J. Cole, M. Rosen, M. Thakar, A. Ambinder, J. Donaldson, A. E. DeZern, K. Cravero, D. Chu, R. Madero-Marroquin, A. Pandey,

P. J. Hurley, J. Lauring, B. H. Park, Hotspot SF3B1 mutations induce metabolic reprogramming and vulnerability to serine deprivation. *J. Clin. Invest.* **129**, 4708–4723 (2019).

37. R. Vivet-Noguer, M. Tarin, C. Canbezdi, S. Dayot, L. Silva, A. Houy, S. Martineau, V. Mieulet, G. Gentric, D. Loew, B. Lombard, F. Nemati, S. Richon, L. Guyonnet, V. Servois, S. Vagner, M. H. Stern, S. Roman-Roman, S. Alsafadi, Glycolysis Dependency as a Hallmark of SF3B1-Mutated Cells. *Cancers* **14**, 2113 (2022).

38. B. Xu, S. Wang, R. Li, K. Chen, L. He, M. Deng, V. Kannappan, J. Zha, H. Dong, W. Wang, Disulfiram/copper selectively eradicates AML leukemia stem cells in vitro and in vivo by simultaneous induction of ROS-JNK and inhibition of NF-κB and Nrf2. *Cell Death Dis.* **8**, e2797–e2797 (2017).

39. S. J. O'Day, A. M. M. Eggermont, V. Chiarion-Sileni, R. Kefford, J. J. Grob, L. Mortier, C. Robert, J. Schachter, A. Testori, J. Mackiewicz, P. Friedlander, C. Garbe, S. Ugurel, F. Collichio, W. Guo, J. Lufkin, S. Bahcall, V. Vukovic, A. Hauschild, Final results of phase III SYMMETRY study: Randomized, double-blind trial of elesclomol plus paclitaxel versus paclitaxel alone as treatment for chemotherapy-naive patients with advanced melanoma. *J. Clin. Oncol.* **31**, 1211–1218 (2013).

40. D. Hedley, A. Shamas-Din, S. Chow, D. Sanfelice, A. C. Schuh, J. M. Brandwein, M. D. Seftel, V. Gupta, K. W. L. Yee, A. D. Schimmer, A phase I study of elesclomol sodium in patients with acute myeloid leukemia. *Leuk. Lymphoma* **57**, 2437–2440 (2016).

41. B. J. Monk, J. T. Kauderer, K. M. Moxley, A. J. Bonebrake, S. B. Dewdney, A. A. Secord, F. R. Ueland, C. M. Johnston, C. Aghajanian, A phase II evaluation of elesclomol sodium and weekly paclitaxel in the treatment of recurrent or persistent platinum-resistant ovarian, fallopian tube or primary peritoneal cancer: An NRG oncology/gynecologic oncology group study. *Gynecol. Oncol.* **151**, 422–427 (2018).

42. M. Mego, D. Svetlovska, D. Angelis V, K. Kalavska, P. Lesko, M. Makovník, J. Obertova, Z. Orszaghova, P. Palacka, M. Rečková, K. Rejlekova, S. M. Z, J. Mardiak, M. Chovanec, Phase

II study of disulfiram and cisplatin in refractory germ cell tumors. The GCT-SK-006 phase II trial. *Invest. New Drugs* **40**, 1080–1086 (2022).

43. K. C. Kelley, K. F. Grossman, M. Brittain-Blankenship, K. M. Thorne, W. L. Akerley, M. C. Terrazas, K. M. Kosak, K. M. Boucher, S. S. Buys, K. A. McGregor, T. L. Werner, N. Agarwal, J. R. Weis, S. Sharma, J. H. Ward, T. P. Kennedy, D. W. Sborov, P. J. Shami, A Phase 1 dose-escalation study of disulfiram and copper gluconate in patients with advanced solid tumors involving the liver using S-glutathionylation as a biomarker. *BMC Cancer* **21**, 510–8 (2021).

44. J. Huang, R. Chaudhary, A. L. Cohen, K. Fink, S. Goldlust, J. Boockvar, P. Chinnaiyan, L. Wan, S. Marcus, J. L. Campian, A multicenter phase II study of temozolomide plus disulfiram and copper for recurrent temozolomide-resistant glioblastoma. *J. Neurooncol* **142**, 537–544 (2019).

45. A. Berkenblit, J. P. Eder Jr, D. P. Ryan, M. V. Seiden, N. Tatsuta, M. L. Sherman, T. A. Dahl, B. J. Dezube, J. G. Supko, Phase I clinical trial of STA-4783 in combination with paclitaxel in patients with refractory solid tumors. *Clin. Cancer Res.* **13**, 584–590 (2007).

46. H. Döhner, A. H. Wei, F. R. Appelbaum, C. Craddock, C. D. DiNardo, H. Dombret, B. L. Ebert, P. Fenaux, L. A. Godley, R. P. Hasserjian, R. A. Larson, R. L. Levine, Y. Miyazaki, D. Niederwieser, G. Ossenkoppele, C. Röllig, J. Sierra, E. M. Stein, M. S. Tallman, H. F. Tien, J. Wang, A. Wierzbowska, B. Löwenberg, Diagnosis and management of AML in adults: 2022 recommendations from an international expert panel on behalf of the ELN. *Blood* **140**, 1345–1377 (2022).

47. L. Wang, M. S. Lawrence, Y. Wan, P. Stojanov, C. Sougnez, K. Stevenson, L. Werner, A. Sivachenko, D. S. DeLuca, L. Zhang, W. Zhang, A. R. Vartanov, S. M. Fernandes, N. R. Goldstein, E. G. Folco, K. Cibulskis, B. Tesar, Q. L. Sievers, E. Shefler, S. Gabriel, N. Hacohen, R. Reed, M. Meyerson, T. R. Golub, E. S. Lander, D. Neuberg, J. R. Brown, G. Getz, C. J. Wu, SF3B1 and other novel cancer genes in chronic lymphocytic leukemia. *N. Engl. J. Med.* **365**, 2497–2506 (2011).

48. M. M. Patnaik, T. L. Lasho, C. M. Finke, C. A. Hanson, J. M. Hodnefield, R. A. Knudson, R. P. Ketterling, A. Pardanani, A. Tefferi, Spliceosome mutations involving SRSF2, SF3B1, and U2AF35 in chronic myelomonocytic leukemia: Prevalence, clinical correlates, and prognostic relevance. *Am. J. Hematol.* **88**, 201–206 (2013).

49. S. J. Furney, M. Pedersen, D. Gentien, A. G. Dumont, A. Rapinat, L. Desjardins, S. Turajlic, S. Piperno-Neumann, P. de la Grange, S. Roman-Roman, M. H. Stern, R. Marais, SF3B1 mutations are associated with alternative splicing in uveal melanoma. *Cancer Discov.* **3**, 1122–1129 (2013).

50. J. W. Harbour, E. D. O. Roberson, H. Anbunathan, M. D. Onken, L. A. Worley, A. M. Bowcock, Recurrent mutations at codon 625 of the splicing factor SF3B1 in uveal melanoma. *Nat. Genet.* **45**, 133–135 (2013).

51. S. L. Maguire, A. Leonidou, P. Wai, C. Marchiò, C. K. Y. Ng, A. Sapino, A. V. Salomon, J. S. Reis-Filho, B. Weigelt, R. C. Natrajan, SF3B1 mutations constitute a novel therapeutic target in breast cancer. *J. Pathol.* **235**, 571–580 (2015).

52. Y. Kong, M. Krauthammer, R. Halaban, Rare SF3B1 R625 mutations in cutaneous melanoma. *Melanoma Res.* **24**, 332–334 (2014).

53. C. Moison, J. F. Spinella, J. Chagraoui, V. P. Lavallée, B. Lehnertz, C. Thiollier, I. Boivin, N. Mayotte, T. MacRae, A. Marinier, J. Hébert, G. Sauvageau, HMGA2 expression defines a subset of human AML with immature transcriptional signature and vulnerability to G2/M inhibition. *Blood Adv.* **6**, 4793–4806 (2022).

54. M. R. Wieckowski, C. Giorgi, M. Lebiedzinska, J. Duszynski, P. Pinton, Isolation of mitochondria-associated membranes and mitochondria from animal tissues and cells. *Nat. Protoc.* **4**, 1582–1590 (2009).

55. A. M. Bolger, M. Lohse, B. Usadel, Trimmomatic: A flexible trimmer for Illumina sequence data. *Bioinformatics* **30**, 2114–2120 (2014).

56. A. Dobin, C. A. Davis, F. Schlesinger, J. Drenkow, C. Zaleski, S. Jha, P. Batut, M. Chaisson, T. R. Gingeras, STAR: Ultrafast universal RNA-seq aligner. *Bioinformatics* **29**, 15–21 (2013).

57. B. Li, C. N. Dewey, RSEM: Accurate transcript quantification from RNA-Seq data with or without a reference genome. *BMC Bioinformatics.* **12**, 323–16 (2011).

58. M. E. Ritchie, B. Phipson, D. Wu, Y. Hu, C. W. Law, W. Shi, G. K. Smyth, limma powers differential expression analyses for RNA-sequencing and microarray studies. *Nucleic Acids Res.* **43**, e47 (2015).

59. S. Shen, J. W. Park, Z. X. Lu, L. Lin, M. D. Henry, Y. N. Wu, Q. Zhou, Y. Xing, rMATS: Robust and flexible detection of differential alternative splicing from replicate RNA-Seq data. *Proc. Natl. Acad. Sci. U.S.A.* **111**, E5593–601 (2014).

60. W. Li, J. Köster, H. Xu, C. H. Chen, T. Xiao, J. S. Liu, M. Brown, X. S. Liu, Quality control, modeling, and visualization of CRISPR screens with MAGeCK-VISPR. *Genome Biol.* **16**, 281–13 (2015).

61. H. Döhner, E. Estey, D. Grimwade, S. Amadori, F. R. Appelbaum, T. Büchner, H. Dombret, B. L. Ebert, P. Fenaux, R. A. Larson, R. L. Levine, F. Lo-Coco, T. Naoe, D. Niederwieser, G. J. Ossenkoppele, M. Sanz, J. Sierra, M. S. Tallman, H. F. Tien, A. H. Wei, B. Löwenberg, C. D. Bloomfield, Diagnosis and management of AML in adults: 2017 ELN recommendations from an international expert panel. *Blood* **129**, 424–447 (2017).

62. C. Fellmann, T. Hoffmann, V. Sridhar, B. Hopfgartner, M. Muhar, M. Roth, D. Y. Lai, I. A. M. Barbosa, J. S. Kwon, Y. Guan, N. Sinha, J. Zuber, An optimized microRNA backbone for effective single-copy RNAi. *Cell Rep.* **5**, 1704–1713 (2013).

63. X. Zhang, A. H. Smits, G. B. A. van Tilburg, H. Ovaa, W. Huber, M. Vermeulen, Proteome-wide identification of ubiquitin interactions using UbIA-MS. *Nat. Protoc.* **13**, 530–550 (2018).

64. C. von Mering , L. J. Jensen, B. Snel, S. D. Hooper, M. Krupp, M. Foglierini, N. Jouffre, M. A. Huynen, P. Bork, STRING: Known and predicted protein-protein associations, integrated and transferred across organisms. *Nucleic Acids Res.* **33**, D433–7 (2005).
